# Key Components of Different Plant Defense Pathways Are Dispensable for Powdery Mildew Resistance of the Arabidopsis *mlo2 mlo6 mlo12* Triple Mutant

**DOI:** 10.3389/fpls.2017.01006

**Published:** 2017-06-19

**Authors:** Hannah Kuhn, Justine Lorek, Mark Kwaaitaal, Chiara Consonni, Katia Becker, Cristina Micali, Emiel Ver Loren van Themaat, Paweł Bednarek, Tom M. Raaymakers, Michela Appiano, Yuling Bai, Dorothea Meldau, Stephani Baum, Uwe Conrath, Ivo Feussner, Ralph Panstruga

**Affiliations:** ^1^Unit of Plant Molecular Cell Biology, Institute for Biology I, RWTH Aachen University Aachen, Germany; ^2^Department of Plant-Microbe Interactions, Max Planck Institute for Plant Breeding Research Cologne, Germany; ^3^Plant-Microbe Interactions, Department of Biology, Faculty of Science, Utrecht University Utrecht, Netherlands; ^4^Plant Breeding, Wageningen University and Research Wageningen, Netherlands; ^5^Department of Plant Biochemistry, Albrecht von Haller Institute, Georg August University Göttingen Göttingen, Germany; ^6^Department of Plant Physiology, Institute for Biology III, RWTH Aachen University Aachen, Germany; ^7^Department of Plant Biochemistry, Göttingen Center for Molecular Biosciences, Georg August University Göttingen Göttingen, Germany

**Keywords:** powdery mildew, MLO, tryptophan, indole glucosinolates, camalexin, jasmonic acid, plant defense, microarray analysis

## Abstract

Loss of function mutations of particular plant *MILDEW RESISTANCE LOCUS O* (*MLO*) genes confer durable and broad-spectrum penetration resistance against powdery mildew fungi. Here, we combined genetic, transcriptomic and metabolomic analyses to explore the defense mechanisms in the fully resistant *Arabidopsis thaliana mlo2 mlo6 mlo12* triple mutant. We found that this genotype unexpectedly overcomes the requirement for indolic antimicrobials and defense-related secretion, which are critical for incomplete resistance of *mlo2* single mutants. Comparative microarray-based transcriptome analysis of *mlo2 mlo6 mlo12* mutants and wild type plants upon *Golovinomyces orontii* inoculation revealed an increased and accelerated accumulation of many defense-related transcripts. Despite the biotrophic nature of the interaction, this included the non-canonical activation of a jasmonic acid/ethylene-dependent transcriptional program. In contrast to a non-adapted powdery mildew pathogen, the adapted powdery mildew fungus is able to defeat the accumulation of defense-relevant indolic metabolites in a MLO protein-dependent manner. We suggest that a broad and fast activation of immune responses in *mlo2 mlo6 mlo12* plants can compensate for the lack of single or few defense pathways. In addition, our results point to a role of Arabidopsis MLO2, MLO6, and MLO12 in enabling defense suppression during invasion by adapted powdery mildew fungi.

## Introduction

The powdery mildew disease, caused by obligate biotrophic ascomycete fungi, leads to enormous agricultural yield losses every year (Dean et al., 2012; Savary et al., 2012). Control of the disease is mediated by application of fungicides and/or genetically encoded types of resistance. Besides typically short-lived isolate-specific immunity, loss-of-function mutations in specific members of the *MILDEW RESISTANCE LOCUS O* (*MLO*) gene family provide stable protection against these pathogens (Jørgensen, 1992; Brown, 2015; Kusch et al., 2016). This durable, broad-spectrum type of immunity is conserved amongst monocots and dicots, including several important crop plants (Jørgensen, 1992; Bai et al., 2007; Humphry et al., 2011; Wang et al., 2014; Berg et al., 2015; Acevedo-Garcia et al., 2016; Kusch et al., 2016; Kusch and Panstruga, 2017). Employment of *mlo* mutant plants in agriculture was pioneered by mutations in a single *Mlo* gene in diploid barley (Jørgensen, 1992). Recent developments in targeted breeding and gene editing enable the transfer of this trait to further important crop plants with more complex genetic features that are less likely to acquire natural mutations in *MLO* genes (Wang et al., 2014; Acevedo-Garcia et al., 2016). However, despite the great potential that interference with MLO protein function offers to agriculture, the biochemical basis of the resistance is still incompletely understood.

In Arabidopsis, three MLO proteins, MLO2 (At2g11310), MLO6 (At1g61560) and MLO12 (At2g39200), cooperatively mediate susceptibility to powdery mildew fungi (Consonni et al., 2006). The strongest susceptibility defect is caused by mutation of *MLO2*, while *MLO6* and *MLO12* have no detectable effect when mutated individually. Double mutant combinations of *mlo2* with *mlo6* or *mlo12* gradually increase the resistance phenotype of the *mlo2* single mutant, and lack of all three proteins results in complete penetration resistance (Consonni et al., 2006). Although *MLO2, MLO6*, and *MLO12* show functional overlap in terms of mediating powdery mildew susceptibility, they seem to be only partially redundant as indicated by their differential quantitative impact on penetration resistance and distinct expression patterns (Arabidopsis eFP Browser (http://bar.utoronto.ca/efp_arabidopsis/cgi-bin/efpWeb.cgi); (Chen et al., 2006; Consonni et al., 2006; Winter et al., 2007; Bhardwaj et al., 2011). Consequently, components that have been recognized as contributors to *mlo2*-mediated resistance have to be validated for their impact on complete penetration resistance of the *mlo2 mlo6 mlo12* triple mutant.

In Arabidopsis, mutation of components of nonhost resistance (NHR) abrogates penetration resistance of *mlo2* mutant plants. The two defense pathways associated with the PENETRATION 1 (PEN1; At3g11820) and PEN2/PEN3 (At2g44490/At1g59870) proteins are major factors of NHR and critical for *mlo2*-mediated resistance (Assaad et al., 2004; Consonni et al., 2006; Stein et al., 2006; Bednarek et al., 2009). *PEN1/SYNTAXIN OF PLANTS (SYP)121* encodes a SOLUBLE *N*-ETHYLMALEMIDE-SENSITIVE FACTOR ATTACHMENT PROTEIN RECEPTOR (t-SNARE) potentially involved in the exocytosis of defense compounds (Kwon et al., 2008; Kwaaitaal et al., 2010). Notably, the situation in Arabidopsis is reminiscent of barley, where the t-SNARE Ror2, the ortholog of PEN1, is required for full powdery mildew resistance of *mlo* mutants (Collins et al., 2003). The β-thioglucoside glucohydrolase PEN2 (Lipka et al., 2005) and the ABC transporter PEN3 (Stein et al., 2006) contribute to hydrolysis of tryptophan (Trp)-derived indole glucosinolates and putative secretion of downstream products with potential antimicrobial activity (Bednarek et al., 2009; Clay et al., 2009; Lu et al., 2015). Besides PEN2 and PEN3, the cytochrome P450 monooxygenases CYP79B2 (At4g39950) and CYP79B3 (At2g22330) are required for *mlo2*-mediated resistance (Consonni et al., 2010). The two functionally redundant enzymes catalyze the conversion of Trp to indole-3-acetaldoxime (IAOx), a precursor of several indolic metabolites, including the phytoalexin camalexin and indole glucosinolates (Hull et al., 2000; Mikkelsen et al., 2000; Zhao et al., 2002; see also **Figure 6** below).

A further link of the *PEN* genes to *mlo2*-mediated resistance is provided by their coexpression with *MLO2* and a set of genes whose transcripts accumulate after biotic stress and stimulation with microbe-associated molecular patterns (MAMPs) (Humphry et al., 2010). This finding and several commonalities shared between *mlo* phenotypes and NHR in Arabidopsis and barley (Humphry et al., 2006) led to the conclusion that both types of penetration resistance have a mechanistic overlap, with *mlo* mutations potentially mediating an accelerated NHR response effective even against adapted pathogens (Peterhänsel et al., 1997; Trujillo et al., 2004; Humphry et al., 2006).

To further examine the resistance mechanism(s) employed by *mlo2 mlo6 mlo12* mutants, we tested known genetic suppressors of *mlo2* resistance for their impact on *mlo* triple mutant plants. Surprisingly, all known contributors to *mlo2*-mediated resistance were found to be fully dispensable for the *mlo2 mlo6 mlo12* resistance phenotype. To identify defense pathways differentially activated in the *mlo2 mlo6 mlo12* mutant, which might help to identify novel components critical for its resistance phenotype, we performed comparative transcript profiling of triple mutant and wild type plants at early time points upon powdery mildew challenge. Our results point to a strong and accelerated activation of defense-related transcriptional programs in *mlo2 mlo6 mlo12* triple mutant plants, which are consequently able to tolerate the lack of individual or even several key defense components.

## Materials and methods

### Plant material and growth conditions

Arabidopsis (*Arabidopsis thaliana*) wild type Col-0, *mlo2* (*mlo2*-5 and *mlo2*-11; Consonni et al., 2006), *mlo2 mlo6 mlo12* (*mlo2*-5 *mlo6*-2 *mlo12*-1; Consonni et al., 2006), *pen1* (*pen1*-1; Collins et al., 2003), *pen2* (*pen2*-1; Lipka et al., 2005), *pen3* (*pen3-*1; Stein et al., 2006), *cyp79B2 cyp79B3* (Zhao et al., 2002), *ora59* (Zander et al., 2014), and *35S::ORA59* (Pré et al., 2008) were described previously. The *mlo2*-6 *mlo6*-4 *mlo12*-8 mutant was generated in the context of this work. Higher order mutants were generated by crosses using these lines as parents. Homozygous mutants were selected by polymerase chain reaction (PCR) using T-DNA- and gene-specific primer sets as described on the T-DNA Express webpage (http://signal.salk.edu/cgi-bin/tdnaexpress) or by cleaved amplified polymorphic sequence (dCAPS) analysis, according to the parental mutations. Primer sequences are available on request. Plants were soil-grown under controlled conditions at a 10/14 h light/dark cycle at 23°C and 65% relative humidity for 4 to 5 weeks until they had developed eight or nine rosette leaves. Due to higher biomass requirements, 5 to 6-week-old plants were used for analysis of phytohormones and tryptophan-derived compounds. For analysis of gene expression (microarray and quantitative reverse transcriptase PCR (qRT-PCR)) and indole glucosinolates, plants were cultivated in Jiffy pellets (Jiffy Products International AS, Stange, Norway). Before sowing Jiffy pellets were moistened in water supplied with 1 ml/l Wuxal fertilizer (Stender, Schermbeck, Germany).

### Powdery mildews and infection assays

*Golovinomyces orontii* and *Blumeria graminis* f.sp. *hordei* (*Bgh*) K1 (Lipka et al., 2005) as well as *Oidium neolycopersici* (Bai et al., 2007) were used in this study. Powdery mildew infection assays were performed as described previously (Consonni et al., 2006; Bai et al., 2007).

### RNA extraction, cDNA synthesis and qRT-PCR

Isolation of total RNA, cDNA synthesis and qRT-PCR were performed essentially as described previously (Consonni et al., 2010; Kwaaitaal et al., 2011) with the primers listed in Table S3.

### Microarray experiment and data analysis

Powdery mildew-inoculated rosette leaves from 4-week-old plants (three per genotype and time point) were harvested at 0 (prior to inoculation), 8 and 12 hpi and frozen in liquid nitrogen. Total RNA was extracted as mentioned above. Copy RNA (cRNA) was prepared following the manufacturer's instructions (www.affymetrix.com/support/technical/manual/expression_manual.affx). Labeled cRNA transcripts were purified using the sample cleanup module (Affymetrix, Santa Clara, USA). Fragmentation of cRNA transcripts, hybridization, and scanning of the high-density oligonucleotide microarrays (Arabidopsis ATH1 genome array; Affymetrix) were accomplished by the Max Planck Genome Centre (Cologne, Germany; (http://mpgc.mpipz.mpg.de/home/) according to the manufacturer's GeneChip Expression Analysis Technical Manual. Three replicates per time point and genotype were processed. The quality of the data was evaluated at probe level by examining the arrays for spatial effects, distribution of absent and presents calls, and the intensity of spike-in controls. The robust multiarray average procedure (Irizarry et al., 2003) was used to correct for background effects and chip effects and to summarize the probe values into probe set values, resulting in 22,811 normalized expression values per array. The R/Bioconductor (Gentleman et al., 2004) package limma (Smyth, 2005) with the graphical user interface limmaGUI (Wettenhall and Smyth, 2004) was employed to pre-process the raw microarray data. Analysis of variance (ANOVA) in combination with the false discovery rate (FDR) test method were applied to correct for the *P*-values (Benjamini and Hochberg, 1995). Genevestigator V3 (https://genevestigator.com/gv/) was used for meta-analysis of gene expression (Hruz et al., 2008). The microarray data are accessible *via* the NCBI Gene Expression Omnibus (GEO) under accession number GSE98673.

Gene ontology (GO) term analysis and functional classification of genes was performed with the PANTHER™ database version 11.1 (http://pantherdb.org; (Mi et al., 2013, 2016). PANTHER Overrepresentation Test was conducted to identify overrepresented biological process GOs (GO database version 1.2.) against 27352 genes of the Arabidopsis reference genome (version April 2015) with a *P*-value cut-off of *P* ≤ 0.05. Bonferroni correction for multiple testing was applied. PANTHER™ GO slim was used for classification of encoded proteins into PANTHER protein classes.

### Indole glucosinolate extraction and measurements by high-performance liquid chromatography-ultraviolet (HPLC/UV)

For I3G, I3G, RA, and 4MI3G measurements, 4 to 5-week-old soil-grown Arabidopsis plants were either leaf-to-leaf inoculated with *G. orontii*, inoculated with *Bgh*, or kept as non-treated control plants. Powdery mildew-inoculated plants were collected at 8, 16, and 24 hpi and frozen in liquid nitrogen. Non-inoculated control plants were harvested prior to inoculation. Extraction and HPLC analysis of secondary metabolites was performed as previously described (Bednarek et al., 2009; Consonni et al., 2010). Measurements were performed with three independent biological replicates.

### Plant phytohormone, indole-3-carboxylic acid (ICA) and camalexin extraction and measurements by high-performance liquid chromatography-tandem mass spectrometry (HPLC-MS/MS)

For salicylic acid (SA), SA beta-glucoside (SAG), ICA and camalexin measurements 5 to 6-week-old soil-grown Arabidopsis plants were either leaf-to-leaf inoculated with *G. orontii*, mock-inoculated with a non-infected leaf, wounded with forceps, or kept as non-treated control plants. Ten plants/genotype were used for each treatment and each of 6 (SA, SAG, and jasmonic acid (JA)) or 9 (camalexin, ICA) biological replicates. Leaves were sampled 30 min (wounding) or 12 h post treatment (all other treatments) and immediately frozen in liquid nitrogen. Per treatment 100 mg of leaf material were used for extraction as previously described (Matyash et al., 2008; Gleason et al., 2016). Extracted samples were analyzed by HPLC-MS/MS as described before (Iven et al., 2012).

### Ethylene (ET) measurements by gas chromatography (GC)

Leaf discs with 6 mm diameter were prepared from 5 to 6-week-old soil-grown Arabidopsis plants and incubated for ca. 5 h in a Petri dish on a wet Whatman filter paper. The leaf discs were either leaf-to-leaf inoculated with *G. orontii*, mock-inoculated with a non-infected leaf, or kept as non-treated control. Leaf discs were transferred individually to tubes filled with 1.6 or 2 ml of 1% agar supplemented with 20 mM MES (pH 5.7). Non-treated leaf discs on 1.6 ml agar were treated with 400 μl 20 mM MES (pH 5.7) containing either 1 μM nlp24 (prepared as 10 mM stock solution in DMSO) to induce ET accumulation or 0.01% DMSO as mock treatment. Three leaf discs/treatment were measured for each of two independent biological experiment at 12 h (*G. orontii*-inoculated, mock-inoculated, non-inoculated) or 3 h (nlp24-treated, mock-treated) post treatment. ET accumulation was measured by GC of a 1 ml sample from the headspace of tubes (Felix et al., 1999).

### Formaldehyde-assisted isolation of regulatory elements (FAIRE)

FAIRE analysis was performed as described by Louwers et al. (2009) with minor modifications. Rosette leaves of five 4 to 5-week-old soil-grown Arabidopsis plants of each genotype were subjected to formaldehyde cross-linking. Chromatin was isolated and sonicated. 1/8th (100 μl) of the total sample volume was taken as an input sample and de-cross-linked overnight at 65°C while 7/8th (700 μl) were taken as the FAIRE sample and kept overnight at −20°C. DNA was extracted with phenol-chloroform-isoamyl alcohol (25:24:1) as described by Louwers et al. (2009). The DNA was dissolved in 20 μl ddH_2_O at 70°C for 15 min.

Amplicon enrichment of crosslinked vs. non(de)-crosslinked DNA by qPCR was performed for *PDF1.2a* and *PDF1.2b*. Analysis was executed with a Roche (Mannheim, Germany) LightCycler 480 II in triplicate 5 μl reactions in a 384-well plate using 1 μl of 1:2 diluted DNA as a template, 0.25 μM of each primer (listed in Table S3) and Takyon No ROX SYBR Mastermix blue dTTP (Eurogentec, Seraing, Belgium) according to the manufacturer's protocol. Melting curve analysis was performed to confirm amplicon identity. To correct for template quality, Crossing point (Cp) data were normalized to values measured for an amplicon within the *ACTIN 2* ORF for each sample (ΔCp = $\bar{\text{x}}$_Cptarget_ - $\bar{\text{x}}$_CpACTIN2_). Afterwards, FAIRE sample data were normalized to input sample data (ΔΔCp = ΔCp _input_ - ΔCp _FAIRE_). To determine amplicon enrichment of crosslinked vs. non-crosslinked DNA 2^ΔΔCp^ values were calculated. For each data point, the mean and standard error of three independent biological replicates were determined.

### Statistical analysis

To test for statistical significant differences of results generalized linear modeling (GLM) with Poisson or Quasi-Poisson regression was performed with R/Bioconductor (Gentleman et al., 2004; Hawkins, 2014).

### Total protein extraction, SDS-PAGE and immunoblot analysis

Whole rosette leaves of 4-week-old plants were harvested at the indicated time points after powdery mildew inoculation, frozen in liquid nitrogen and homogenized. Total protein extracts were prepared in 120 μl/100 mg fresh weight (FW) lysis buffer (20 mM HEPES pH 7.5; 13% sucrose; 1 mM EDTA; 1 mM DTT; 0.01% Triton X-100, complete Mini protease inhibitor cocktail tablet/10 ml buffer (Roche). Cell debris was removed by centrifugation, the protein concentration was determined by Bradford assay and protein samples were boiled for 10 min in 2x loading buffer. Following gel electrophoresis, proteins were blotted onto nitrocellulose membranes and the transfer visualized with the Ponceau protein dye. Blots were incubated with PEN2 antiserum (1:5,000 dilution in PBS-T at 4°C overnight; Lipka et al., 2005) and subsequently with a horseradish peroxidase-coupled goat anti rabbit secondary antiserum (Santa Cruz Biotechnology, Dallas, USA; 1:5000 dilution in PBS-T). For signal detection, the SuperSignal West Pico solution (Thermo Fisher Scientific, Waltham, USA) was used according to the manufacturer's instructions. Luminescence was documented on X-ray films.

## Results

### Powdery mildew resistance in the *mlo2 mlo6 mlo12* triple mutant functions independently of PEN-mediated defense

**Figure 1:**
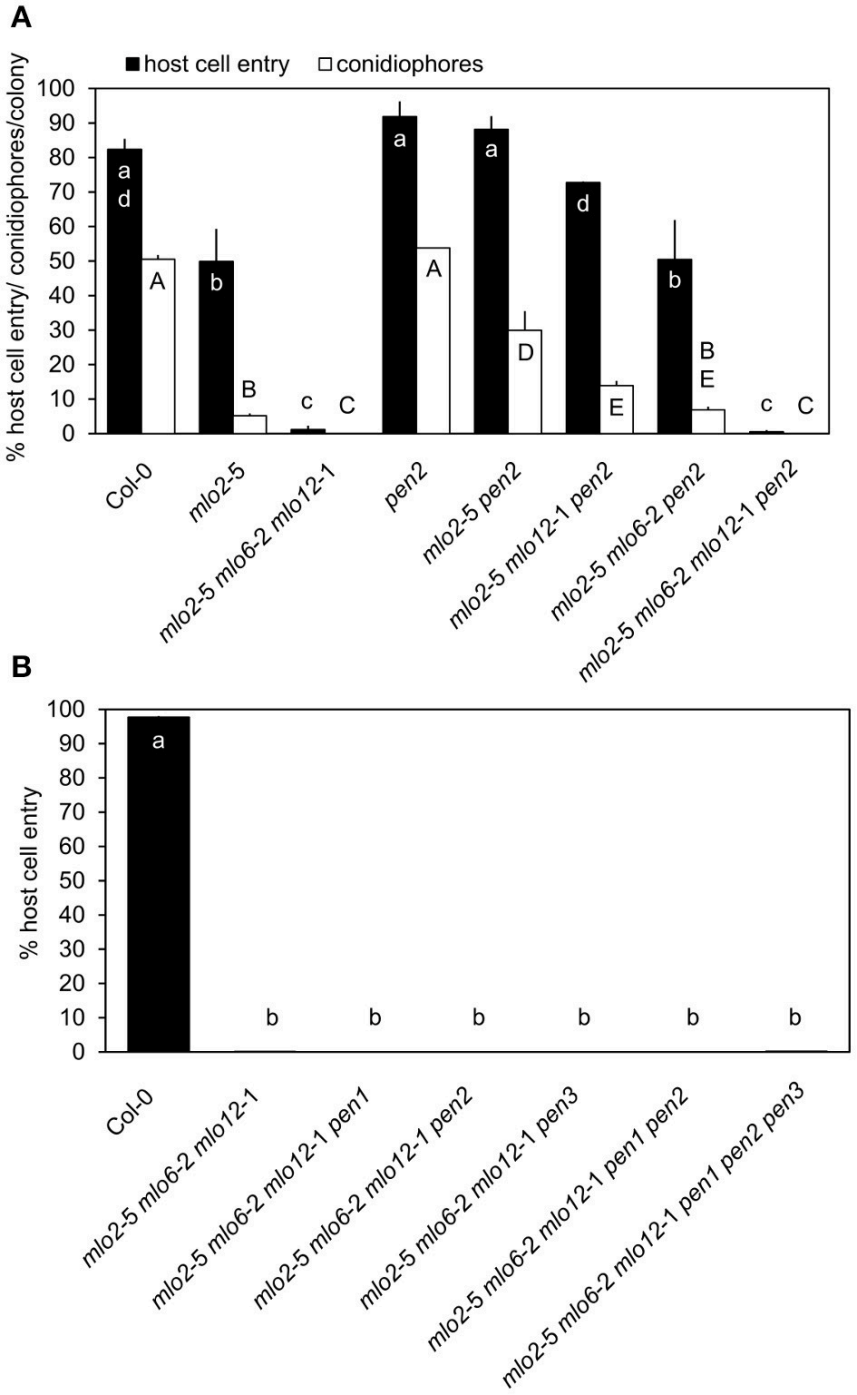
*mlo2 mlo6 mlo12*-mediated resistance functions independently of the PEN1 and PEN2/PEN3 defense pathways. Four to five-week-old plants of the indicated genotypes were inoculated with *G. orontii* and % host cell entry **(A,B)** and conidiophore formation per colony **(A)** were evaluated at 48 hpi or 7 dpi, respectively. Data represent means of 3 independent biological replicates ± SE. Distinct lower-case (% host cell entry) or upper-case (conidiophores/colony) letters indicate statistically significant differences between groups as determined by GLM (*P* ≤ 0.05).

To determine the functional contribution of PEN2 activity to the complete powdery mildew immunity of the *mlo2 mlo6 mlo12* triple mutant, we generated the *mlo2 mlo6 mlo12 pen2* quadruple mutant as well as the *mlo2 mlo6 pen2* and *mlo2 mlo12 pen2* triple mutants and assessed their infection phenotypes with the adapted powdery mildew pathogen *G. orontii* in comparison to a set of informative control plants. Consistent with previous results, the *mlo2 pen2* double mutant displayed restored fungal penetration rates, which resembled the Col-0 wild type and the *pen2* single mutant values (Figure 1A; Consonni et al., 2006, 2010). Interestingly, triple mutant plants defective in *MLO2, PEN2* and either *MLO6* or *MLO12* showed a gradual decrease in *G*. *orontii* host cell invasion and conidiation, which was more pronounced for the *mlo2 mlo6 pen2* than for the *mlo2 mlo12 pen2* triple mutant (Figure 1A). This phenotype is reminiscent of the successive decline of powdery mildew susceptibility observed for the *mlo2 mlo6* and *mlo2 mlo12* double mutants (Consonni et al., 2006). Unexpectedly, the *mlo2 mlo6 mlo12 pen2* quadruple mutant exhibited full resistance against the powdery mildew fungus, which was indistinguishable from the *mlo2 mlo6 mlo12* triple mutant, indicating that powdery mildew resistance in the *mlo2 mlo6 mlo12* mutant is independent of PEN2 activity (Figure 1A). Consistently, also lack of the PEN3 ABC transporter, which presumably acts in the same pathway and potentially exports products of PEN2 (Stein et al., 2006; Lu et al., 2015), was insufficient to overcome penetration resistance in the *mlo2 mlo6 mlo12 pen3* triple mutant (Figure 1B).

We previously demonstrated that *mlo2*-mediated powdery mildew resistance requires the vesicle-dependent secretory pathway involving the t-SNARE PEN1, as the *mlo2 pen1* double mutant exhibits restored powdery mildew penetration rates similar to *mlo2 pen2* plants (Consonni et al., 2006). Therefore, we also tested the functional contribution of *PEN1* to powdery mildew resistance in the context of the *mlo2 mlo6 mlo12* mutant. The *G*. *orontii*-challenged *mlo2 mlo6 mlo12 pen1* quadruple mutant retained complete resistance to the fungus, which was comparable to the *mlo2 mlo6 mlo12* triple and the *mlo2 mlo6 mlo12 pen2* or *mlo2 mlo6 mlo12 pen3* quadruple mutants (Figure 1B). To examine if simultaneous disruption of both defense pathways affects powdery mildew resistance mediated by loss-of-function of *MLO2, MLO6* and *MLO12*, the *mlo2 mlo6 mlo12 pen1 pen2* and the *mlo2 mlo6 mlo12 pen1 pen2 pen3* mutants were generated. Notably, resistance against the pathogen was not altered in the respective quintuple or hexuple mutants (Figure 1B). To exclude that our observations were specific for the Arabidopsis-*G. orontii* pathosystem, we tested the respective quadruple, quintuple and hexuple mutants with a second adapted powdery mildew fungus, *Oidium neolycopersici*, and obtained similar results (Figure S1). Together these data suggest that the *PEN1*- and *PEN2/PEN3*-dependent defense pathways, separately or in combination, are dispensable for powdery mildew resistance in the *mlo2 mlo6 mlo12* triple mutant.

### Transcriptomic analysis indicates accelerated accumulation of defense-related transcripts in the *mlo2 mlo6 mlo12* mutant upon inoculation with *G. orontii*

To elucidate the transcriptional patterns and possibly discover novel components associated with powdery mildew resistance in the *mlo2 mlo6 mlo12* mutant, we performed global gene expression analysis using the Affymetrix ATH1 GeneChip, comparing transcript abundance in the *mlo2 mlo6 mlo12* mutant with Col-0 wild type plants upon inoculation with *G. orontii*. Therefore, we sampled whole rosette leaves from wild type control and mutant plants at 0 (prior to inoculation), 8 and 12 hpi. These early time points were chosen to compare the transcriptional reprogramming events of both genotypes around the time of host cell penetration (Kuhn et al., 2016). At later time points—after fungal penetration of epidermal cells in susceptible Col-0—transcript patterns between mutant and wild type plants are likely to be different simply because of the differential developmental stage of the pathogen on the two genotypes.

**Figure 2:**
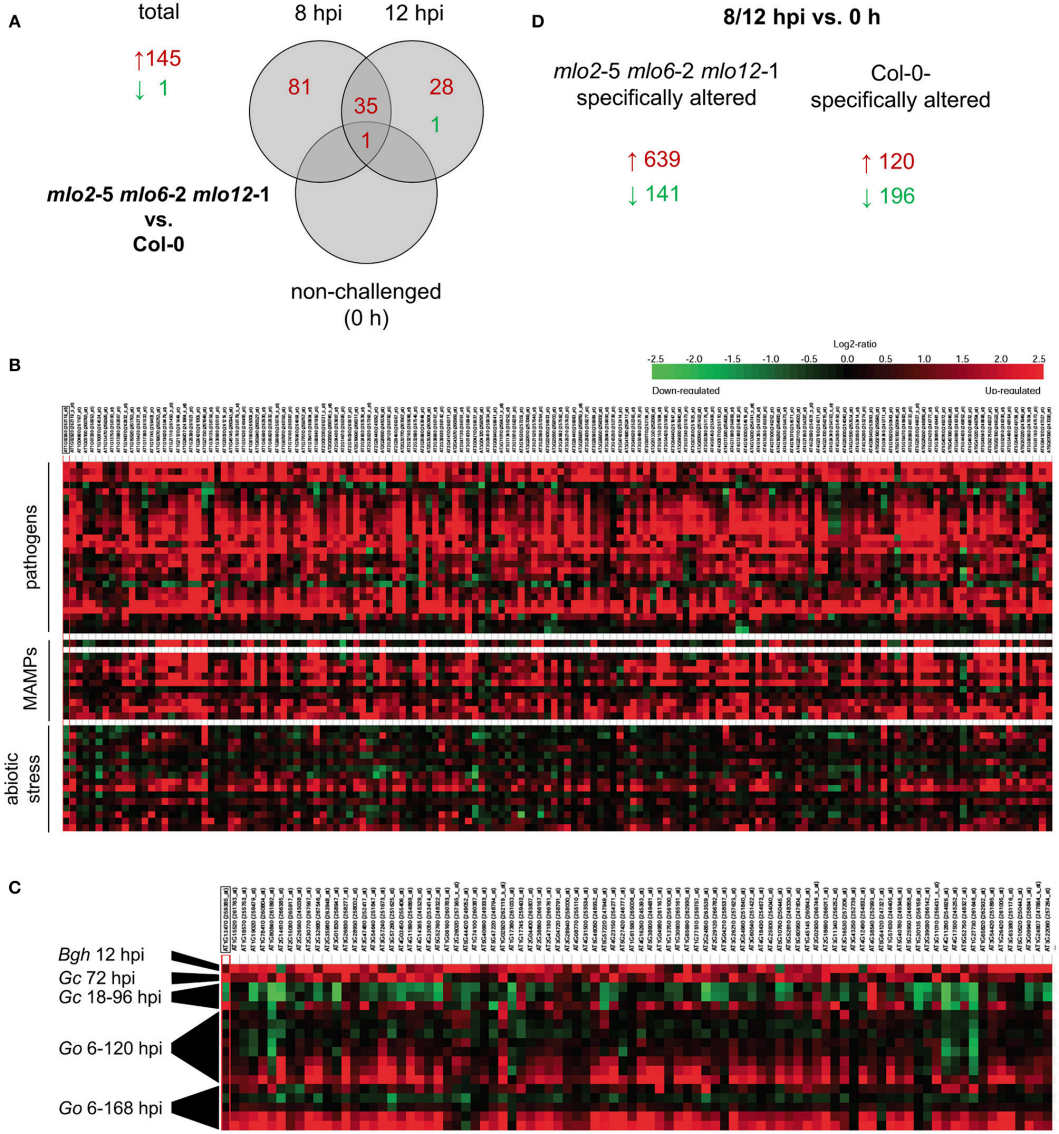
*mlo2 mlo6 mlo12* contributes to accelerated transcript accumulation of (defense) related genes during *G. orontii* infection. Four to five-week-old *mlo2-*5 *mlo6-*2 *mlo12*-1 and Col-0 plants were inoculated with *G. orontii* and sampled prior to inoculation (0 h, non-challenged) or at 8 and 12 hpi for comparative transcriptome analysis using the Affymetrix ATH1 GeneChip. All samples were analyzed in triplicate. **(A)** Total numbers of genes and Venn diagram displaying the number of genes with statistically significantly (*P* ≤ 0.05) increased (≥ 2-fold; red) or decreased (≤ 0.5-fold; green) transcript abundance in *mlo2 mlo6 mlo12* vs. Col-0 plants at the indicated time points after *G. orontii* challenge. **(B)** Heat map of relative expression of genes that were statistically significantly induced (≥ 2-fold; *P* ≤ 0.05) *in mlo2 mlo6 mlo12* vs. Col-0 in selected Genevestigator studies based on the displayed stimuli. **(C)** Heat map of relative expression of genes associated with defense-related GO terms that were statistically significantly induced (≥ 2-fold; *P* ≤ 0.05) *in mlo2 mlo6 mlo12* vs. Col-0 in powdery mildew-related Genevestigator studies (*Bgh, B. graminis* f.sp. *hordei*; *Go, G. orontii* (6, 12, 18, 24, 48, 72, 96, 120 hpi and 6, 24, 72, 120, 168 hpi); *Gc, G. cichoracearum* (18, 36, 96 hpi). **(D)** Number of genes with statistically significantly altered (≥ 2-fold (red) or ≤ 0.5-fold (green); *P* ≤ 0.05 *P* ≤ 0.05) transcript abundance in *mlo2 mlo6 mlo12* or Col-0 at 8 and/or 12 hpi vs. 0 h.

Based on the comparative microarray analysis between genotypes we identified candidate genes with a statistically significant (*P* ≤ 0.05) and at least two-fold change (log_2_ 1-fold change) in transcript abundance in the *mlo2 mlo6 mlo12* mutant compared with Col-0 wild type plants in response to *G. orontii* challenge (Figure 2A, Table S1A). Based on these criteria, sets of 117 and 64 genes (representing 145 unique genes) exhibited elevated transcript levels in the *mlo2 mlo6 mlo12* mutant compared to the wild type at 8 and 12 hpi, respectively (Figure 2A, Table S1A). Thirty-six genes overlapped between both time points and 81 genes had higher transcript levels exclusively at 8 hpi, whereas only 28 genes showed enhanced transcript accumulation specific to the later time point (12 hpi; Figure 2A). These results indicate a more rapid and/or stronger transcriptional activation of comparatively few (145) genes in response to powdery mildew challenge in the *mlo2 mlo6 mlo12* mutant at early stages after *G. orontii* inoculation. Notably, only one gene, *At3g43250*; coding for an uncharacterized protein of 249 amino acids with a domain of unknown function (DUF572) and a putative role in cell cycle regulation, exhibited higher mRNA levels also in non-treated mutant plants at 0 h, suggesting that no major constitutive gene expression occurs in the unchallenged *mlo2 mlo6 mlo12* genotype (Figure 2A). The latter result validates that the *mlo2 mlo6 mlo12* plants did not yet enter the phase of premature leaf senescence, which is characterized by a distinct expression profile in the unchallenged mutant (Consonni et al., 2010). In contrast to the total of 145 genes showing higher transcript abundance in *mlo2 mlo6 mlo12* vs. Col-0, only one gene (*At3g44735*; encoding PHYTOSULFOKINE 3 PRECURSOR (PSK3)) exhibited a statistically significant decrease in transcript levels in the triple mutant compared to the wild type upon challenge with *G. orontii* (Figure 2A, Table S1A). Thus, transcriptional activation rather than inhibition appears to dominate the response of *mlo2 mlo6 mlo12* mutant plants to *G. orontii* inoculation. We therefore further concentrated on the 145 genes with elevated transcript levels in the triple mutant vs. Col-0. *In silico* meta-analysis using Genevestigator V3 (Hruz et al., 2008) indicated that these genes are predominantly induced in response to various pathogens and MAMPs (Figure 2B). By contrast, abiotic stresses, with the exception of osmotic stress and prolonged wounding, generally do not impact the transcript levels of these genes (Figure 2B). Functional classification of the corresponding gene products revealed induction of many transcripts encoding non-classified proteins (27%), followed by oxidoreductases (14%) and transferases (11%; Figure S2A). GO term analysis based on the PANTHER™ database (http://pantherdb.org) revealed a statistically significant overrepresentation of GO terms related to defense, representing 84 genes (Tables S1C, S2). Strikingly, inspection of the Genevestigator expression profiles unveiled that the same transcripts that showed early induction in the *mlo2 mlo6 mlo12* mutant accumulate at much later time points (starting at 72 hpi) in wild type plants inoculated with the adapted powdery mildews *G. orontii* and *G. cichoracearum* (Figure 2C). By contrast, inoculation of Arabidopsis with the non-adapted barley powdery mildew fungus *Bgh* induced transcript accumulation of these genes already at 12 hpi (Figure 2C), suggesting that upon infection by a virulent fungus their expression is either repressed or failed to be elicited during the early interaction—an effect that is overcome by the *mlo2 mlo6 mlo12* mutant.

We further evaluated the microarray data with respect to statistically significant (*P* ≤ 0.05) alterations of transcript abundance over time within one genotype (no comparison to the other genotype). This type of analysis indicated that a greater number of genes were induced more than 2-fold upon inoculation in the *mlo2 mlo6 mlo12* mutant than in Col-0 at 8 hpi (394 vs. 90) or 12 hpi (396 vs. 44) compared to non-inoculated plants (0 h; Figure S2B). In total, 639 unique transcripts accumulated at one or both time points more than 2-fold in the triple mutant but not in the wild type, while 120 showed more than 2-fold higher abundance in Col-0 but not in the *mlo2 mlo6 mlo12* genotype (Figure 2D). Since the differences in transcript levels between wild type and mutant are comparatively small, the majority of these differentially expressed genes were not identified in our more stringent analysis described above (at least 2-fold difference in transcript abundance between genotypes).

Together these data indicate an accelerated responsiveness of genes related to defense in the *mlo2 mlo6 mlo12* mutant upon *G. orontii* challenge. While comparatively few genes (145) exhibit a strong (more than 2-fold) difference in transcript levels between *mlo2 mlo6 mlo12* and Col-0, many more (639) show a minor but mutant-specific increase of mRNA abundance.

### Transcript levels of JA/ET-responsive genes increase more rapidly in the *mlo2 mlo6 mlo12* mutant upon powdery mildew challenge

**Figure 3:**
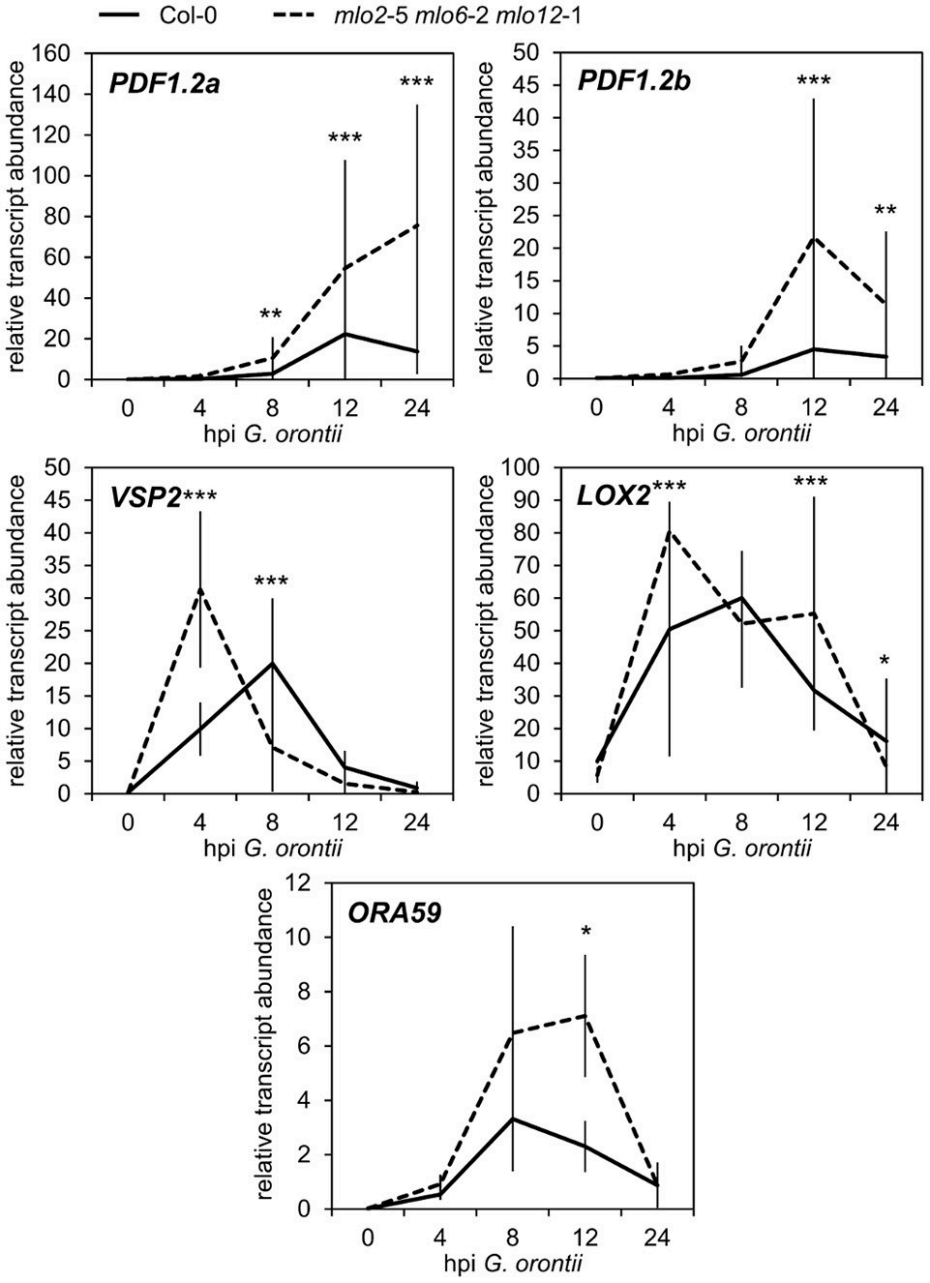
Transcripts of JA-responsive genes accumulate more rapidly and to higher levels in *mlo2 mlo6 mlo12* vs. Col-0 after inoculation with *G. orontii*. qRT-PCR analysis of *PDF1.2a, PDF1.2b, VSP2, LOX2* and *ORA59* transcripts in leaves of 4–5-week-old Col-0 (solid line) and *mlo2 mlo6 mlo12* (dashed line) plants inoculated with *G. orontii* and sampled prior to inoculation (0 h) or at 4, 8, 12, and 24 hpi. Gene expression was normalized to the transcript levels of the reference gene *At4g26420*. Means ± SE of two independent biological replicates are shown. Asterisks indicate a statistically significant difference from Col-0 (^***^P ≤ 0.01, ^**^P ≤ 0.01, ^*^P ≤ 0.05, GLM).

Besides GO terms related to defense, we found JA- and ET-associated GO terms significantly overrepresented among induced transcripts in the *mlo2 mlo6 mlo12* triple mutant after *G. orontii* inoculation (Table S2). By detailed manual inspection of the transcriptomic data we identified genes previously described to be involved in or induced by the JA/ET signaling pathways (Table 1). These include genes encoding members of the APETALA2/ETHYLENE RESPONSE FACTOR (AP2/ERF)-domain transcription factor family (*OCTADECANOID-RESPONSIVE ARABIDOPSIS AP2/ERF 59* (*ORA59*; *At1g06160), ERF1* (*At4g17500*), and *ERF2* (*At5g47220*); (Pré et al., 2008), prominent JA/ET marker genes encoding the plant defensins PDF1.2a (At5g44420) and PDF1.2b (At2g26020; Penninckx et al., 1996, 1998), and further genes known to be induced in wild type plants upon treatment with JA and/or ET (Table 1). Differential transcript accumulation of these genes suggests an increased JA/ET signaling activity in *mlo2 mlo6 mlo12* mutant plants early upon *G. orontii* challenge. We tested this hypothesis experimentally with a focus on *PDF1.2a* and *PDF1.2b* (here collectively referred to as *PDF1.2*) expression. First, we aimed to confirm the elevated *PDF1.2* transcript levels in the *mlo2 mlo6 mlo12* mutant using qRT-PCR in a time-course experiment after *G. orontii* challenge (0, 4, 8, 12, and 24 hpi; Figure 3). In contrast to previously published results obtained with the powdery mildew pathogen *G. cichoracearum* (Zimmerli et al., 2004) but in accordance with our microarray data, *PDF1.2* mRNA abundance was strongly induced in Col-0 wild type plants upon challenge with *G. orontii* (Figure 3; Figures S3A,B). *PDF1.2* transcript levels increased from 8 hpi onwards, peaked at 12 hpi, and were still considerably above background levels at 24 hpi. The *mlo2 mlo6 mlo12* triple mutant exhibited a similar kinetic of *PDF1.2* mRNA accumulation, but revealed markedly higher *PDF1.2* mRNA levels at all three time points (8, 12, and 24 hpi) compared to Col-0 wild type plants (Figure 3; Figures S3A,B). Following challenge with non-adapted *Bgh*, Col-0 wild type plants showed a moderate increase of *PDF1.2* transcript accumulation at 24 hpi, which is consistent with previous data (Zimmerli et al., 2004). Resembling the results obtained with *G. orontii*, this increase was statistically significantly higher in the *mlo2 mlo6 mlo12* mutant compared to Col-0 (Figure S4; compare to Figure 3).

**Table 1:** Transcript levels of JA/ET-responsive genes are elevated in *mlo2 mlo6 mlo12* mutant plants in response to inoculation with the powdery mildew fungus *G. orontii*.

**Arabidopsis Genome ID**	**Gene annotation and putative function of encoded protein**	**Fold change** ***mlo2 mlo6 mlo12*****/Col-0** ***G. orontii***		**Induction of gene expression in Col-0 in response to** ^a^	
		**8 hpi**	**12 hpi**	**JA**	**JA + ET**
*AT5G44420*	*PDF1.2a* (Plant Defensin 1.2a)	5.0	6.3	yes	yes
*AT5G61160*	*AACT1* (Anthocyanin-5-Aromatic Acyltransferase 1)	4.9	6.2	yes	yes
*AT3G49620*	*DIN11* (Dark Inducible 11)	4.7	7.3	yes	yes
*AT3G04720*	*PR4* (Pathogenesis-Related Protein 4)	4.3	2.8	-	yes
*AT4G16260*	Glycosyl hydrolase family 17 protein	4.0	5.8	-	yes
*AT2G26020*	*PDF1.2b* (Plant Defensin 1.2b)	3.9	7.1	yes	yes
*AT2G26560*	*PLP2* (Phospholipase A 2A)	3.5	-	yes	yes
*AT1G67810*	Fe-S metabolism associated domain-containing protein	3.5	2.7	-	yes
*AT3G16530*	Lectin like protein	3.2	2.2	yes	yes
*AT1G26380*	FAD-binding domain-containing protein	2.9	-	yes	yes
*AT5G25250*	Unknown protein	2.8	2.2	-	yes
*AT1G17745*	*PGDH* (3-Phosphoglycerate Dehydrogenase)	2.7	2.5	yes	yes
*AT3G28930*	*AIG2*, avrRpt2-induced gene	2.6	1.7	yes	yes
*AT2G38860*	Protease I (pfpI)-like protein YLS5	2.5	2.0	-	yes
*AT1G06160*	*ORA59* (AP2/ERF domain transcription factor)	2.4	2.8	-	yes
*AT1G02920*	*GST7* (Glutathione-S-Transferase 7)	2.4	-	yes	yes
*AT2G04400*	*IGPS* (Indole-3-Glycerol Phosphate Synthase)	2.3	2.0	-	yes
*AT5G47220*	*ERF2* (AP2/ERF domain transcription factor)	2.2	1.9	yes^b^	yes^b^
*AT5G10520*	*RBK1* (ROP binding protein kinases 1)	-	3.8	-	yes
*AT1G30135*	*JAZ8* (Jasmonate-Zim-domain protein 8)	-	2.7	yes^b^	-^b^
*AT1G07260*	*UGT71C3* (UDP-glycosyltransferase 71C3)	1.3	2.6	-	yes
*AT4G08770*	Peroxidase, putative	1.7	2.6	yes	yes
*AT4G24350*	Phosphorylase family protein	-	2.3	yes	yes
*AT1G51760*	*JR3* (Jasmonic Acid Responsive 3)	-	2.3	yes^b^	-^b^
*AT4G17500*	*ERF1* (AP2/ERF domain transcription factor 1)	-	2.2	yes^b^	yes^b^
*AT4G24340*	Phosphorylase family protein	-	2.3	yes	yes
*AT5G54960*	*PDC2* (Pyruvate Decarboxylase 2)	-	2.2	-	yes
*AT1G17380*	*JAZ5* (Jasmonate-Zim-domain protein 5)	-	2.2	yes^b^	-^b^
*AT4G34200*	*EDA9* (Embryo Sac Development Arrest 9)	-	2.0	yes	yes

a *Genes induced in Col-0 wild type plants after treatment with JA or JA and ET (Pré et al., 2008)*.

b *These genes were characterized using Genevestigator V3 (Hruz et al., 2008)*.

*Genes were identified by comparative transcriptome analysis upon G. orontii challenge and manually selected from candidate genes with a statistically significant (P ≤ 0.05) and at least two-fold change in transcript abundance in the mlo2-5 mlo6-2 mlo12-1 mutant compared with Col-0 wild type (Table S1). This gene selection was compared with published expression data from Col-0 plants treated with JA or JA and ET*.

In addition to *PDF1.2*, also the genes *VEGETATIVE STORAGE PROTEIN 2* (*VSP2*; *At5g24770*) and *LIPOXYGENASE 2* (*LOX2*; *At3g45140*) are generally highly expressed in response to JA signaling activity (Bell and Mullet, 1993; Benedetti et al., 1995). Therefore, we analyzed the effect of *G. orontii* inoculation on the transcript levels of these additional two JA marker genes in *mlo2 mlo6 mlo12* mutant plants. mRNA abundance of both *VSP2* and *LOX2* quickly and transiently increased in wild type and *mlo2 mlo6 mlo12* mutant plants upon challenge with *G. orontii* and peaked at 4–8 hpi (Figure 3; Figures S3C,D). Similar to *PDF1.2*, transcript levels of *VSP2* and *LOX2* were higher in the *mlo2 mlo6 mlo12* mutant (starting at 4 hpi) compared to wild type upon *G. orontii* inoculation. However, mRNA abundance of these two genes was similar for Col-0 and *mlo2 mlo6 mlo12* at later time points and therefore followed a different pattern than *PDF1.2*.

The AP2/ERF transcription factor ORA59, which was transcriptionally induced in *mlo2 mlo6 mlo12* upon infection by *G. orontii* (Table 1), acts as a master regulator of JA/ET signaling by integrating both pathways (reviewed in Pieterse et al., 2009). ORA59 is required for JA- and ET-induced transcription of *PDF1.2*, and, together with ERF1, activates the expression of genes related to biosynthesis of Trp and Trp-derived indolic compounds [Lorenzo et al., 2003; Pré et al., 2008 (Supplemental Table 1)]. Analysis of *ORA59* transcript abundance by qRT-PCR confirmed that the gene was induced in both, Col-0 and *mlo2 mlo6 mlo12* plants, upon *G. orontii* infection. In contrast to *PDF1.2, VSP2*, and *LOX2, ORA59* mRNA levels reached statistically significantly (*P* ≤ 0.05) higher levels in mutant than in wild type plants only at later time points (12 hpi) during infection (Figure 3, Figure S3E). Taken together, the different expression patterns of these genes suggest that distinct factors contribute to their regulation in the context of powdery mildew challenge.

### ORA59 is dispensable for *mlo2 mlo6 mlo12*-mediated resistance

**Figure 4:**
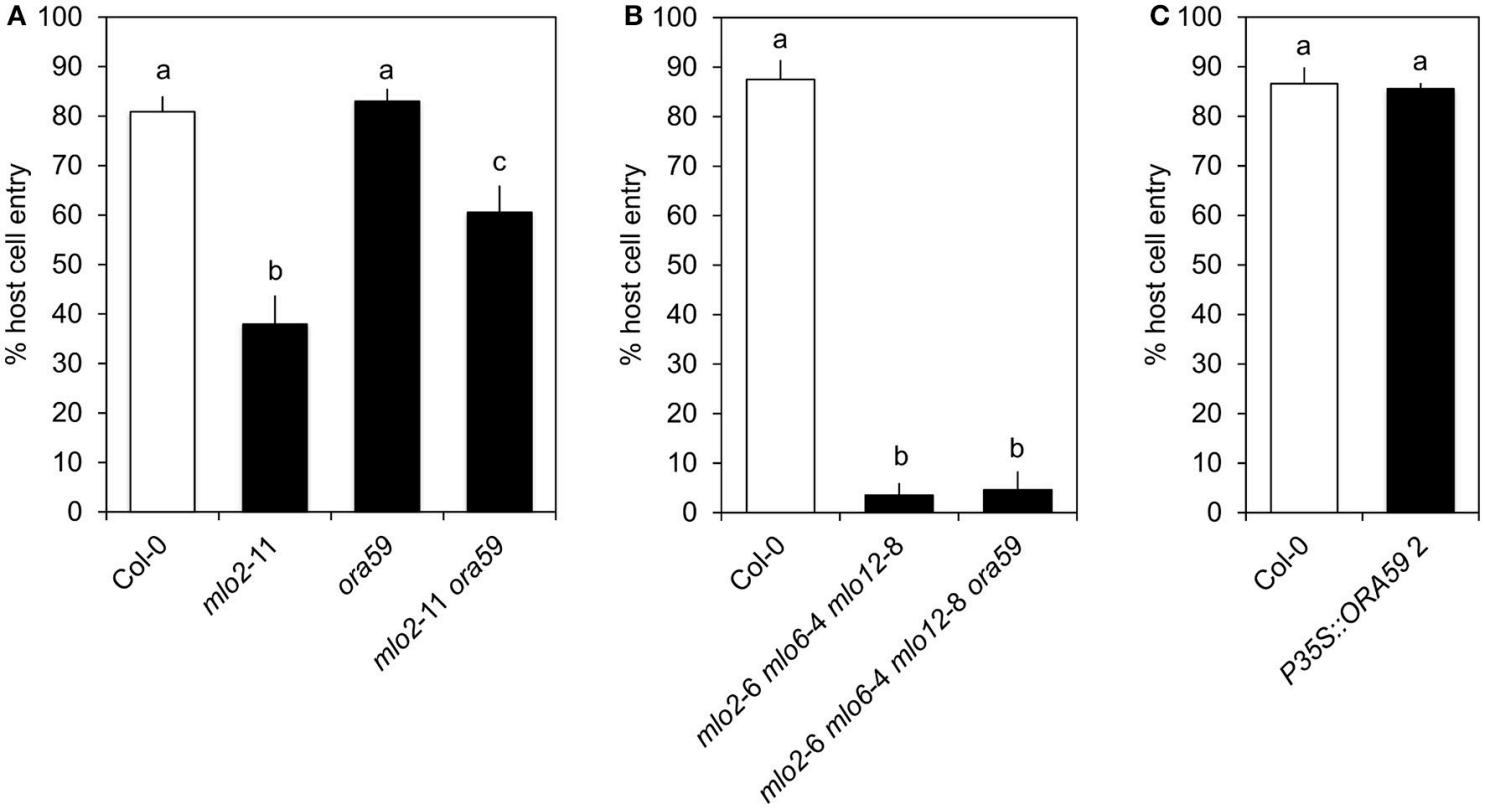
*ORA59* contributes to *mlo2*- but is not required for *mlo2 mlo6 mlo12*-mediated resistance against *G. orontii*. Four to five-week-old plants of indicated genotypes were inoculated with *G. orontii* and % host cell entry was evaluated at 48 hpi. Data represent means of 5 **(A)** or 3 **(B,C)** independent biological replicates ± SE and five plants/biological replicate. Distinct lower-case letters indicate statistically significant differences between groups as determined by GLM (*P* ≤ 0.05).

As mentioned above, ORA59 strongly contributes to expression of JA/ET-responsive genes in the context of Col-0 wild type plants. To test whether ORA59 is required for *mlo*-mediated resistance, we quantified penetration success of *G. orontii* on the *mlo2 ora59* double mutant. In contrast to other mutants of JA/ET signaling components (Consonni et al., 2006), mutation of *ORA59* in an *mlo2* background increased host cell entry of the virulent fungus from ca. 40 to ca. 60% (Figure 4A). However, additional mutation of *mlo6* and *mlo12* abrogated this effect (Figure 4B). Moreover, mutation or overexpression of *ORA59* in a wild type background did not impact susceptibility against *G. orontii* (Figure 4C, Figure S5). In conclusion, *ORA59* does not generally contribute to basal resistance of wild type plants against *G. orontii*. Still, contribution of ORA59 to *mlo2*-mediated resistance of Arabidopsis against *G. orontii* suggests a protective effect of JA/ET signaling in plants lacking MLO2. However, although JA/ET-responsive genes are more rapidly induced in *mlo2 mlo6 mlo12* vs. wild type plants (Table 1 and Table S2), this requirement is overcome by the *mlo* triple mutant. This fact suggests that increased abundance of typically ORA59-dependent transcripts such as *PDF1.2* is not critical for *mlo2 mlo6 mlo12*-mediated penetration resistance.

### The more rapid accumulation of *PDF1.2* transcripts in *mlo2 mlo6 mlo12* is not due to an open chromatin state of the *PDF1.2* promoters

An enhanced induction of gene expression after a pathogen stimulus is also a hallmark of defense priming associated with systemic acquired resistance (SAR; Mur et al., 1996; Kohler et al., 2002; Beckers et al., 2009). This phenomenon correlates with histone modifications that are associated with increased accessibility of the respective defense gene promoters (Jaskiewicz et al., 2011). To investigate whether *mlo2 mlo6 mlo12* mutant plants are constitutively primed for intense expression of JA/ET-related genes, we tested promoter accessibility of *PDF1.2* in naïve, unchallenged plants by formaldehyde-assisted isolation of regulatory elements (FAIRE). The relative enrichment of promoter-localized amplicons of both genes (*PDF1.2a* and *PDF1.2b*) in crosslinked and non-crosslinked samples from two distinct *mlo2 mlo6 mlo12* mutant allele combinations did not differ from wild type (Figure S6). This finding suggests that the *cis*-regulatory chromatin regions of *PDF1.2* have similar nucleosome density in *mlo2 mlo6 mlo12* and Col-0 plants. Consequently, the early *PDF1.2* transcript accumulation observed for *mlo2 mlo6 mlo12* compared with Col-0 plants seems not to be due to increased accessibility of the respective promoters.

### Col-0 and *mlo2 mlo6 mlo12* plants exhibit similar JA, ET, and salicylic acid (SA) levels upon challenge with *G. orontii*

**Figure 5:**
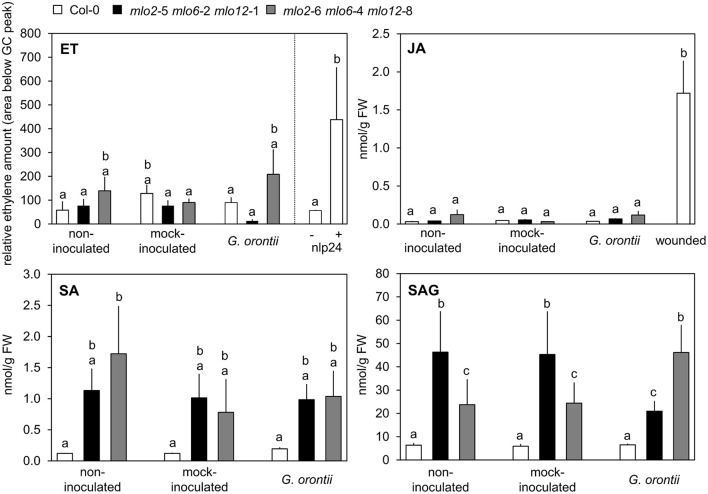
Levels of defense hormones are not differentially impacted by *G. orontii* inoculation in *mlo2 mlo6 mlo12* vs. Col-0 but SA accumulates in *mlo2 mlo6 mlo12* independently of an infection. Five to six-week-old plants of indicated genotypes were leaf-to-leaf inoculated with *G. orontii*, mock-inoculated, treated with 1 μM nlp24 (ET), wounded (JA), or kept as non-inoculated control plants. At 12 hpi, 3 h post nlp24 treatment or 30 min after wounding leaves were sampled and hormones were measured by GC (ET) or HPLC-MS/MS (JA, SA, SAG). Data represent means of 2 (ET) or 6 (JA, SA, SAG) biological replicates +/− SE. Distinct lower-case letters indicate statistically significant differences between groups as determined by GLM (*P* ≤ 0.05).

As our microarray data revealed an overrepresentation of JA/ET-responsive genes in the *mlo2 mlo6 mlo12* triple mutant after *G. orontii* inoculation, we next assessed if levels of JA and ET were altered in the mutant compared to the Col-0 wild type during fungal penetration (12 hpi). ET accumulation was unaltered in both genotypes (Figure 5), and also JA concentrations were low in wild type and mutant plants with or without powdery mildew or mock inoculation with no statistically significant differences. By contrast, treatment with the necrosis- and ethylene-inducing peptide 1 (Nep1)-like protein (NLP) epitope nlp24 (Oome et al., 2014) or wounding of leaves caused accumulation of ET and JA, respectively (Figure 5). In summary, the data suggest that JA and ET levels are not differentially altered in *mlo2 mlo6 mlo12* mutant and Col-0 wild type plants in response to *G. orontii*.

In addition to a well-described antagonistic action of SA on JA signaling, evidence accumulates that moderately enhanced SA levels can also act synergistically on expression of JA-responsive genes in Arabidopsis (Mur et al., 2006; Liu et al., 2016). *mlo2 mlo6 mlo12* mutants show a developmentally controlled increase in SA levels starting at approximately 6 weeks after sowing (Consonni et al., 2006). However, accumulation of SA in Arabidopsis *mlo* mutants in response to powdery mildew infection has not been assessed yet. To investigate whether powdery mildew infection is able to further raise SA levels in *mlo2 mlo6 mlo12* mutants we determined SA abundance in 5 to 6-week-old plants following challenge with *G. orontii* (12 hpi; Figure 5). We noted an increased accumulation of free and conjugated SA (the storage form salicylic acid beta-glucoside (SAG)) in *mlo2 mlo6 mlo12* mutants compared to Col-0 at this time point. Nevertheless, *G. orontii* inoculation failed to further heighten SA and/or SAG levels (Figure 5). This excludes a promoting effect of SA on the infection-specific early expression of JA/ET-responsive genes in *mlo2 mlo6 mlo12*.

### Transcript levels of indole metabolite biosynthesis genes are induced to higher levels in the *mlo2 mlo6 mlo12* mutant upon powdery mildew challenge

**Figure 6:**
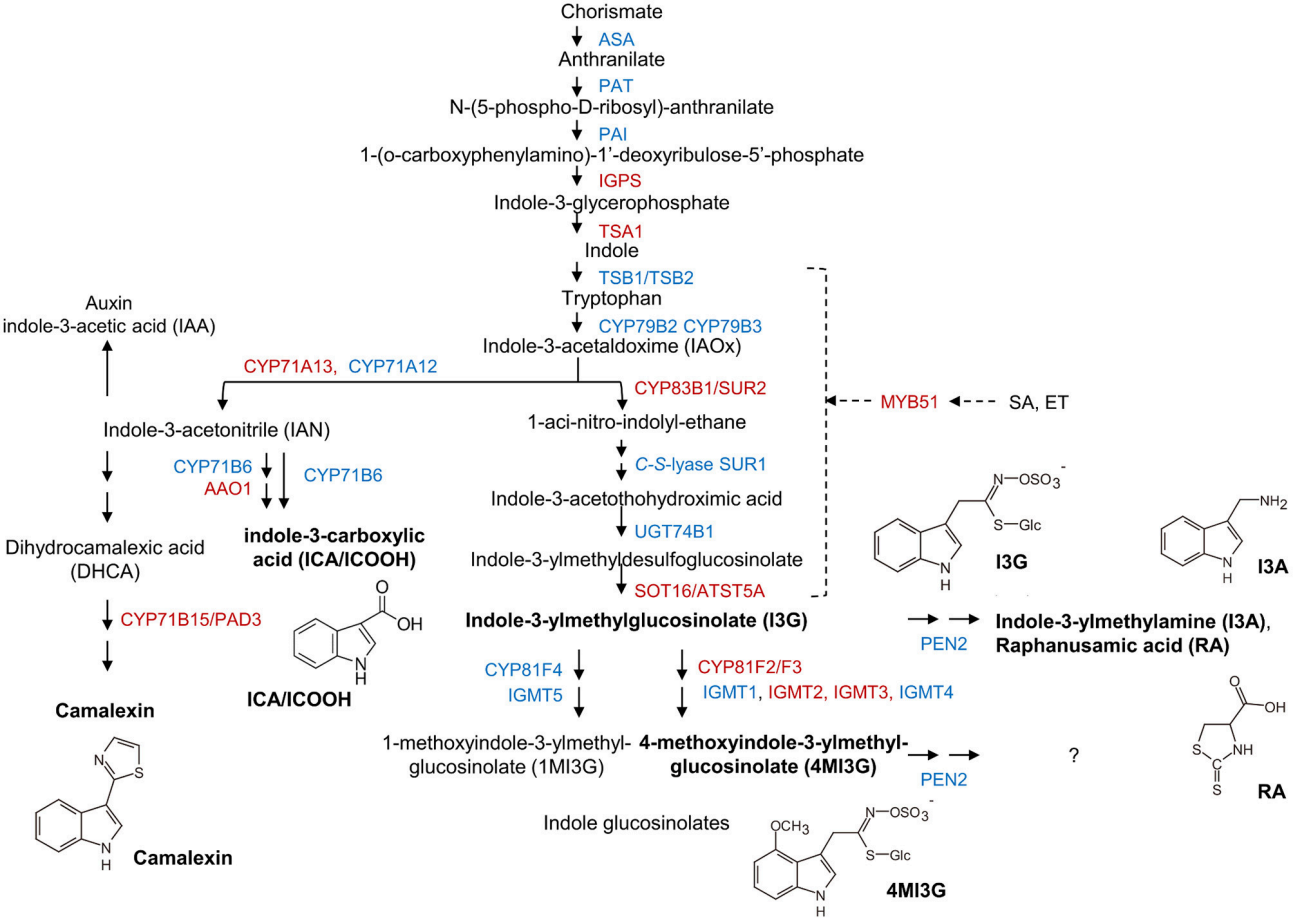
Transcripts of biosynthetic genes of the Trp/indole glucosinolate and camalexin pathways accumulate in *mlo2 mlo6 mlo12*. Scheme of Trp, camalexin, indole-3-carboxylic acid (ICA) and indole glucosinolate biosynthesis/metabolism. Proteins with increased (red) or unaltered (blue) transcript accumulation in *mlo2 mlo6 mlo12* vs. wild type following *G. orontii* challenge are indicated. Formulae of subsequently analyzed substances (bold) are shown. Trp is synthesized from chorismate and subsequently hydrolyzed to indole-3-acetaldoxime (IAOx) by the cytochrome P450 monooxygenases CYP79B2 and CYP79B3. IAOx is either converted to camalexin, ICA, auxin or indolic glucosinolates. PEN2 is an atypical myrosinase that hydrolyzes 4MI3G for antifungal defense. MYB51 regulates expression of several key enzymes of indole glucosinolate biosynthesis.

Besides GO terms related to JA/ET signaling, also indole glucosinolate biosynthesis-associated terms were significantly overrepresented among genes that were induced in *mlo2 mlo6 mlo12* vs. wild type (Table 2). Detailed analysis revealed that mRNAs of several genes encoding Trp biosynthetic and metabolic enzymes hyper-accumulated in the *mlo2 mlo6 mlo12* mutant upon inoculation with *G. orontii* (Table 2, Figure 6). Examples are genes encoding the Trp biosynthesis enzymes TRYTOPHAN SYNTHASE ALPHA SUBUNIT (TSA1; At3g54640) as well as INDOLE-3-GLYCEROL PHOSPHATE SYNTHASE (IPGS; At2g04400). Moreover, genes coding for several cytochrome P450 monooxygenases that catalyze dedicated steps of indole glucosinolate and camalexin biosynthesis were found to be induced by *G. orontii* in *mlo2 mlo6 mlo12* mutant plants (Table 2, Figure 6). The gene encoding transcription factor MYB51 (At1g18570), a key regulator of genes encoding indole glucosinolate biosynthesis enzymes, was also present among these genes (Gigolashvili et al., 2007). Together these findings emphasize an important role for Trp-derived indolic secondary metabolites in *mlo*-mediated powdery mildew defense, which is in agreement with a requirement of these metabolites for resistance in the *mlo2* single mutant (Consonni et al., 2010).

**Table 2:** Transcripts of biosynthesis genes of tryptophan-related antimicrobials hyperaccumulate in the *mlo2 mlo6 mlo12* mutant in response to inoculation with *G. orontii*.

**Arabidopsis Genome ID**	**Gene annotation and putative function of encoded protein**	**Process**	**Fold change** ***mlo2 mlo6 mlo12*****/Col-0 (*****G. orontii*****)**		***P*** **-value**	
			**8 hpi**	**12 hpi**	**8 hpi**	**12 hpi**
*AT2G04400*	*IGPS* (Indole-3-Glycerol Phosphate Synthase)	Trp synthesis	2.3	2.0	6.2E^−05^	3.6E^−04^
*AT3G54640*	*TSA1* (Tryptophan Synthase Alpha subunit 1)	Trp synthesis	2.7	2.5	4.8E^−05^	8.9E^−05^
*AT5G57220*	*CYP81F2* (Cytochrome P450 monooxygenase)	IG synthesis	2.3	1.5	2.0E^−02^	2.1E^−01^
*AT4G31500*	*CYP83B1* (Cytochrome P450 monooxygenase)	IG synthesis	2.1	1.6	4.5E^−03^	5.5E^−02^
*AT1G18570*	*MYB51* (MYB domain transcription factor 51)	IG synthesis	2.5	2.0	8.0E^−03^	3.6E^−02^
*AT1G74100*	*SOT16* (Sulfotransferase 16)	IG synthesis	2.0	1.8	6.8E^−03^	1.6E^−02^
*AT2G30770*	*CYP71A13* (Cytochrome P450)	Camalexin, ICA synthesis	6.4	4.0	7.3E^−05^	1.0E^−03^
*AT3G26830*	*CYP71B15/PAD3* (Phytoalexin Deficient 3, Cytochrome P450 monooxygenase)	Camalexin synthesis	6.1	2.3	2.3E^−04^	4.2E^−02^
*AT1G21120*	*IGMT2* (Indole glucosinolate o-methyltransferase 2)	IG synthesis	2.7	1.3	7.1E^−03^	4.3E^−01^
*AT5G20960*	*AAO1* (Aldehyde oxidase 1)	ICA synthesis	2.5	1.9	1.6E^−03^	1.8E^−02^

*Genes were identified by comparative transcriptome analysis upon G. orontii challenge and manually selected from candidate genes with a statistically significant (P ≤ 0.05) and at least two-fold change in transcript abundance in the mlo2-5 mlo6-2 mlo12-1 mutant compared with Col-0 (Table S1). Trp, tryptophan; IG, indole glucosinolate; ICA, indol-3-carboxylic acid*.

### The phytoalexin camalexin hyperaccumulates in *mlo2 mlo6 mlo12* during *G. orontii* infection

**Figure 7:**
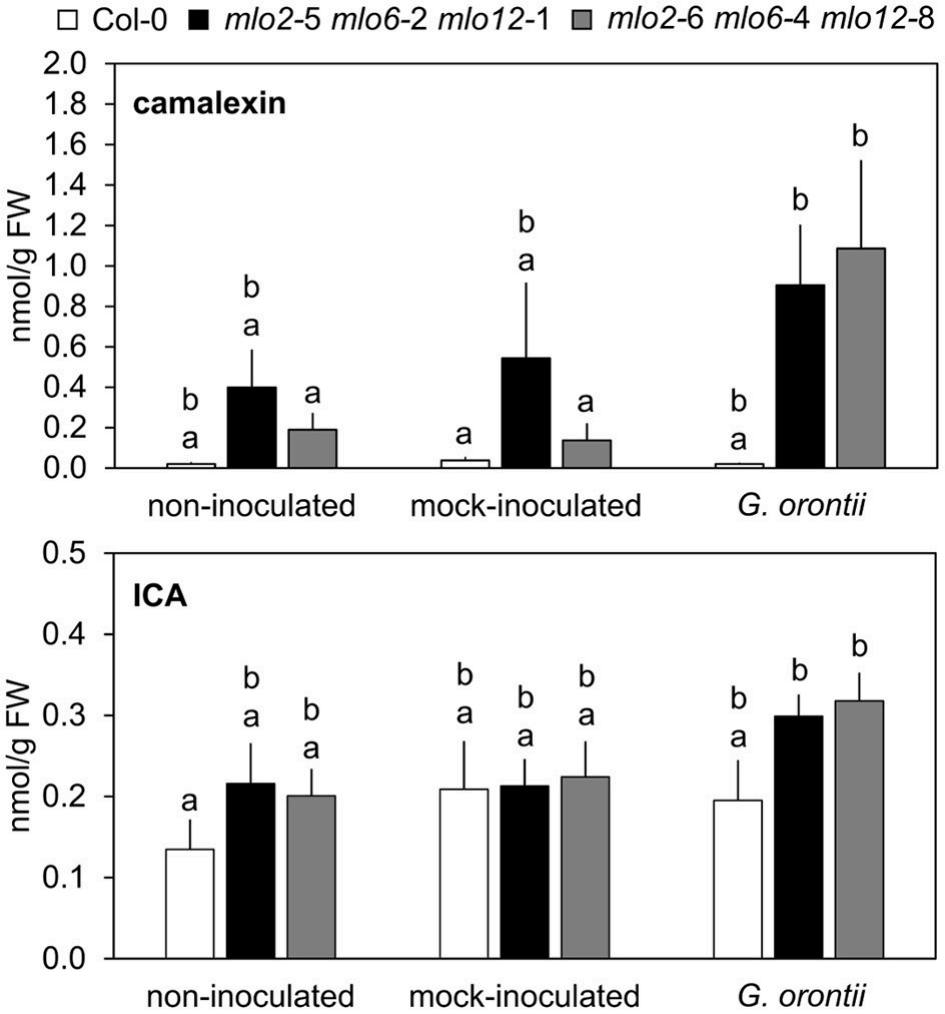
Camalexin but not ICA hyperaccumulates in *mlo2 mlo6 mlo12* plants during *G. orontii* infection. Five to six-week-old plants of indicated genotypes were leaf-to-leaf inoculated with *G. orontii*, mock-inoculated or kept as non-inoculated control plants. At 12 hpi the leaves were sampled, metabolites extracted and compounds measured by HPLC-MS/MS. Data represent means of 9 biological replicates ± SE. Distinct lower-case letters indicate statistically significant differences between groups as determined by GLM (*P* ≤ 0.05).

The elevated transcript levels of genes encoding components for the biosynthesis of Trp-derived secondary metabolites in the *mlo2 mlo6 mlo12* mutant (see above and Table 2) might translate into elevated accumulation of such metabolites in powdery mildew-challenged triple mutant plants. To examine this possibility, we established a comparative metabolite profile of Col-0 wild type and *mlo2 mlo6 mlo12* mutant plants following powdery mildew inoculation. Since we observed an increased transcript abundance of *CYP71A13* (*At2g30770*) and *CYP71B15/PAD3* (*At3g26830*), two genes encoding cytochrome P450 monooxygenases involved in biosynthesis of the phytoalexin camalexin (Figure 6, Table 2), we tested for abundance of camalexin in unchallenged, mock- and *G. orontii*-inoculated plants at 12 h after the respective treatment. In all three conditions we observed an increased abundance of camalexin in two different *mlo2 mlo6 mlo12* triple mutants compared to wild type (Figure 7). This result is consistent with our previous findings that naïve *mlo2 mlo6 mlo12* plants accumulate camalexin in a developmentally controlled manner (Consonni et al., 2010). Furthermore, powdery mildew-challenged *mlo2 mlo6 mlo12* mutants accumulated more camalexin than mock-treated or Col-0 control plants (Figure 7). By contrast, abundance of indole-3-carboxylic acid (ICA), which derives from the same indolic precursor as camalexin (indole-3-acetonitrile/IAN; Figure 6; (Böttcher et al., 2014), was largely unaltered between mock- and *G. orontii*-inoculated plants (Figure 7). This finding is in agreement with our microarray data, which revealed unaltered transcript abundance of the ICA biosynthesis gene *CYP71B6* (*At2g24180*) in the mutant vs. wild type after *G. orontii* inoculation.

### MLO2, MLO6, and MLO12 mediate suppression of 4MI3G accumulation in the compatible powdery mildew interaction

**Figure 8:**
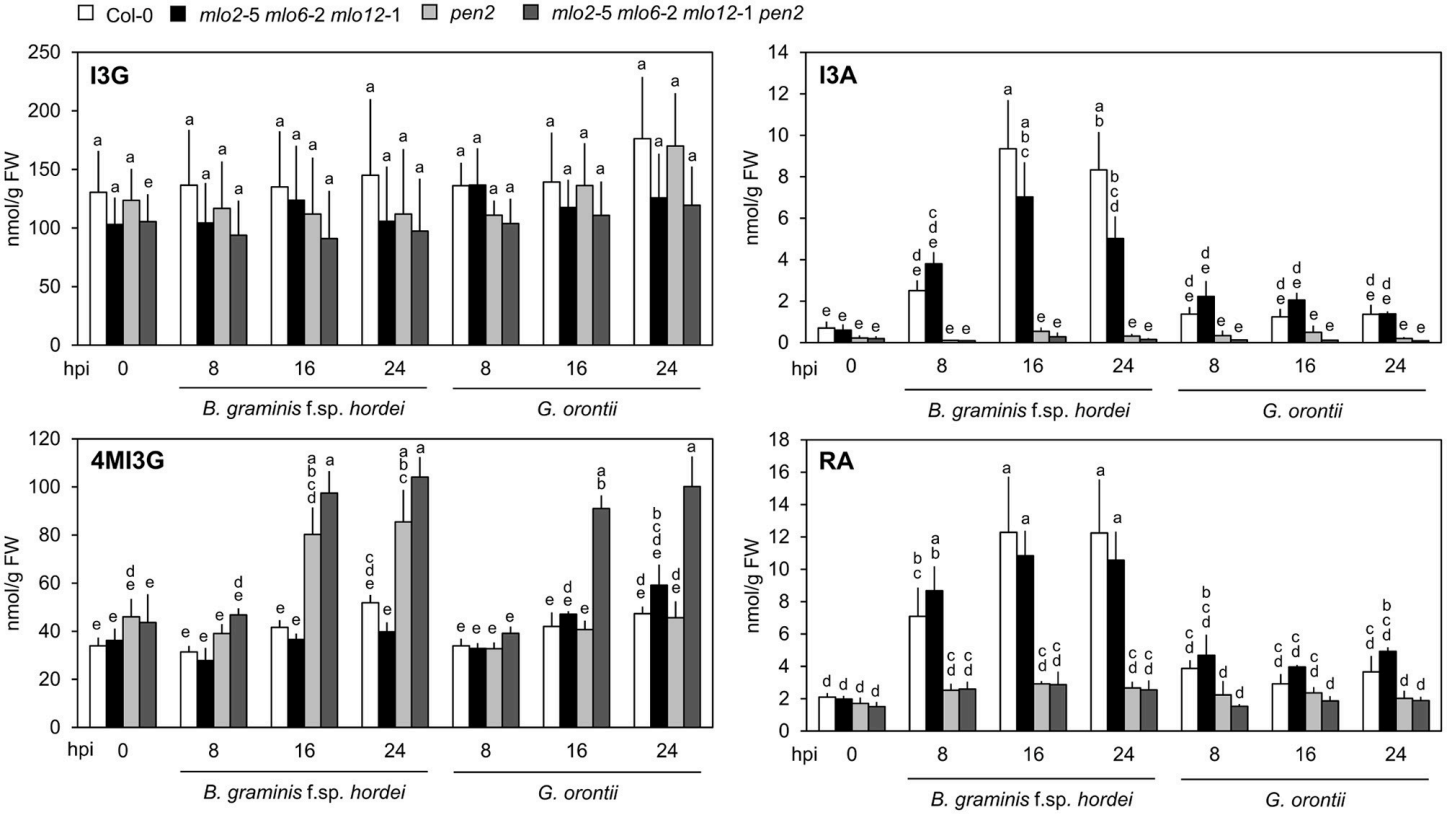
Accumulation of 4MI3G upon inoculation depends on the presence of MLO2, MLO6 and MLO12 during interaction with an adapted, but not with a non-adapted powdery mildew fungus. Four to five-week-old plants of the indicated genotypes were inoculated with *G. orontii, B. graminis* f. sp. *hordei*, or kept as non-inoculated control plants. Leaves were sampled at the indicated time points after inoculation, metabolites extracted and compound concentrations measured by HPLC. Data represent means of 3 independent biological replicates ± SE. Distinct lower-case letters indicate statistically significant differences between groups as determined by GLM (*P* ≤ 0.05).

It was previously hypothesized that 4-methoxyindol-3-ylmethyl glucosinolate (4MI3G) is the biologically-relevant PEN2 substrate leading to the formation of yet unknown toxic compounds that are important for the restriction of non-adapted powdery mildews (Bednarek et al., 2009). 4MI3G is synthesized by CYP81F2/F3 (At5g57220/At4g37400), INDOLE GLUCOSINOLATE *O*-METHYLTRANSFERASE (IGMT)1 (At1g21100) and IGMT2 (At1g21120) from the precursor indol-3-ylmethylglucosinolate (I3G). I3G also serves as a substrate for PEN2, which catalyzes its conversion into indol-3-ylmethylamine (I3A) and raphanusamic acid (RA; Figure 6; Bednarek et al., 2009). We determined I3G, I3A, RA and 4MI3G levels in the Col-0 wild type, *pen2, mlo2 mlo6 mlo12* triple and *mlo2 mlo6 mlo12 pen2* quadruple mutant genotypes at 0 (prior to inoculation), 8, 16, and 24 h after challenge with the adapted or non-adapted powdery mildews *G. orontii* and *Bgh*, respectively (Figure 8). While I3G levels were not significantly altered by any of the treatments, I3A and RA accumulated after inoculation with the non-adapted fungus *Bgh* in dependence of PEN2, as previously shown by Bednarek et al. (2009) for 16 hpi. By contrast, *G. orontii* inoculation did not trigger an increase in the levels of these compounds. Consistent with its turnover by PEN2 (Bednarek et al., 2009), we observed that 4MI3G levels did not rise in Col-0 and *mlo2 mlo6 mlo12* triple mutant plants at any time point after inoculation with either the adapted or non-adapted powdery mildew pathogen (Figure 8). By contrast, mutants lacking a functional PEN2 myrosinase showed increased 4MI3G levels after treatment with the non-adapted fungus *Bgh*. This *Bgh*-induced 4MI3G accumulation was solely dependent on the *pen2* mutation and independent of the presence or absence of functional MLO proteins. Interestingly, 4MI3G accumulation in response to *G. orontii* was not observed in *pen2* single mutants, suggesting that the adapted powdery mildew pathogen either fails to induce additional 4MI3G biosynthesis or interferes with its accumulation. Strikingly, quadruple mutants defective in *PEN2* and the three *MLO* genes exhibited higher 4MI3G levels after *G. orontii* challenge, indicating that the adapted powdery mildew fungus is principally able to induce 4MI3G accumulation. The differential 4MI3G quantities cannot be ascribed to altered amounts of PEN2, since PEN2 protein levels were comparable in Col-0 and *mlo2 mlo6 mlo12* mutant plants upon challenge with *Bgh* or *G. orontii* (Figure S7). Thus, *G. orontii* may either be able to metabolize 4MI3G or to suppress its biosynthesis, a process which seems to be dependent on the presence of the three MLO proteins.

### Indolic metabolites are entirely dispensable for powdery mildew resistance in the *mlo2 mlo6 mlo12* mutant

Mutants defective in *PEN2* are unable to hydrolyze the indolic intermediates I3G and 4MI3G, which are the supposed precursors of secreted antifungal metabolites (Bednarek et al., 2009). However, *pen2* mutants are still able to accumulate a wide range of additional indolic metabolites, some of which have proven (e.g., camalexin) or suspected antimicrobial activity (Bednarek, 2012). Penetration resistance of *mlo2* depends on the functionally redundant CYP79B2 and CYP79B3 monooxygenases, which catalyze the first dedicated step in the biosynthesis of indolic metabolites (Figure 6; Hull et al., 2000; Mikkelsen et al., 2000). Respective *cyp79B2 cyp79B3* double mutants are consequently depleted of Trp-derived indolics (Zhao et al., 2002).

**Figure 9:**
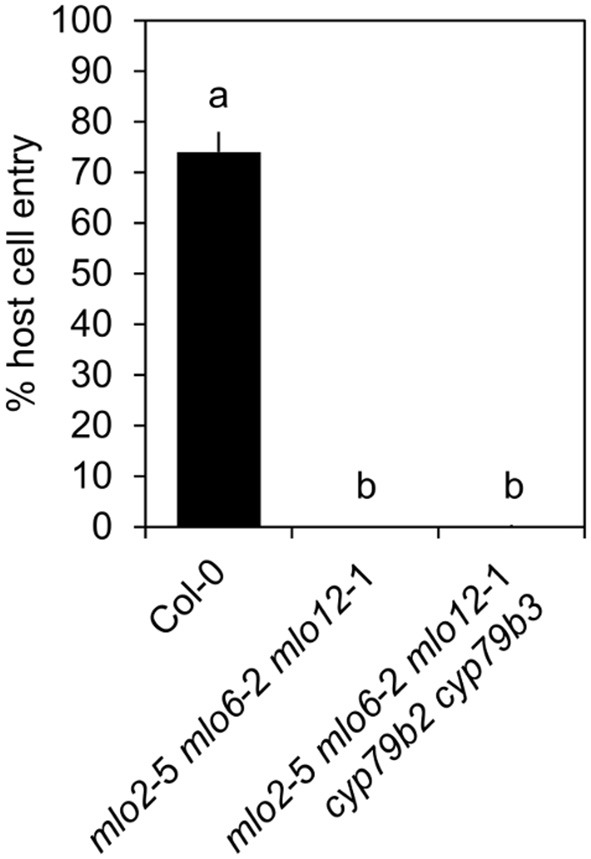
Indolic secondary metabolites are dispensable for powdery mildew resistance in the *mlo2 mlo6 mlo12* triple mutant. Four to five-week-old plants of the indicated genotypes were inoculated with *G. orontii* and % host cell entry was evaluated at 48 hpi. Data represent means of 3 independent biological replicates ± SE. Distinct lower-case letters indicate statistically significant differences between groups as determined by GLM (*P* ≤ 0.05).

Upregulation of camalexin and indole glucosinolate biosynthesis genes and accumulation of the respective compounds in *mlo2 mlo6 mlo12* vs. wild type suggests that these might collectively contribute to the increased resistance of the mutant. To test whether resistance of the *mlo2 mlo6 mlo12* triple mutant relies on the constitutive or pathogen-induced accumulation of other indolic metabolites, we generated the *mlo2 mlo6 mlo12 cyp79B2 cyp79B3* quintuple mutant. Analysis of *G. orontii*-challenged plants revealed that this mutant, similar to the *mlo2 mlo6 mlo12 pen1, mlo2 mlo6 mlo12 pen2, mlo2 mlo6 mlo12 pen3, mlo2 mlo6 mlo12 pen1 pen2* mutants and the *mlo2 mlo6 mlo12 pen1 pen2 pen3* mutants, retains complete resistance to the powdery mildew pathogen (Figure 9; see also Figure 1). In sum our genetic data indicate that powdery mildew resistance in the *mlo2 mlo6 mlo12* mutant does not require the biosynthesis of antimicrobial indolic metabolites.

## Discussion

### Presence of MLO2, MLO6, and MLO12 retards induction of defense-related genes during powdery mildew infection

Performing comparative analysis of microarray data, Genevestigator studies and investigation of GO term overrepresentation, we found that the *mlo2 mlo6 mlo12* mutant shows an accelerated/increased accumulation of transcripts associated with defense, JA/ET signaling and biosynthesis of indole-derived antimicrobials at early time points after *G. orontii* inoculation (Figure 2, Tables S1, S2). The finding is reminiscent of the results of a previous study, which used a cDNA array to probe differences in transcript accumulation between powdery mildew-challenged barley wild type (*Mlo* genotype) and *mlo* mutant plants (Zierold et al., 2005). In this work, the authors used RNA from epidermal cells for hybridization to the array and found that ca. 10% of the genes (311 out of 3128) were reproducibly upregulated in response to attack by *Bgh*, with the *mlo* genotype typically showing a more pronounced differential regulation (Zierold et al., 2005). Since we deployed whole rosette leaves as the biological source material for our transcript profiling experiments, we currently do not know whether the observed alterations in transcript patterns derive solely from the epidermis (the primary target tissue of powdery mildew fungi) or whether they represent a systemic response in the entire leaf. Experiments based on laser capture micro-dissection recently revealed distinctive transcript accumulation at powdery mildew infection sites in Arabidopsis (Chandran et al., 2010). The lack of a constitutively altered transcript profile and boosted expression of many genes in the *mlo2 mlo6 mlo12* genotype hint at a strong impact of quantitative or kinetic rather than qualitative alterations contributing to the powdery mildew phenotype. A fast and robust induction of multiple plant immune pathways could explain why mutations of *PEN* genes in various combinations are insufficient to break resistance in *mlo2 mlo6 mlo12* plants (Figure 1, Figure S1). This contrasts the situation for *mlo2* mutants, in which qualitative changes in single defense pathways, such as PEN1-mediated secretion or PEN2/PEN3-mediated biosynthesis and delivery of glucosinolate-derived antimicrobials, strongly contribute to the resistance phenotype (Consonni et al., 2006). Interestingly, according to our microarray data, all three *PEN* genes are induced in *mlo2 mlo6 mlo12* and wild type Col-0 plants at 8 and 12 h after inoculation with *G. orontii* (Table S1B). In conclusion, while the two pathways represented by PEN1 and PEN2/PEN3 are generally involved in powdery mildew resistance, their necessity is overcome in *mlo2 mlo6 mlo12* mutant plants.

### Transcript accumulation of JA/ET-responsive genes in the *mlo2 mlo6 mlo12* mutant upon powdery mildew challenge occurs independently of altered JA or ET levels

JA/ET-related defense has preferentially been linked to resistance against necrotrophic parasites (Thomma et al., 2001; Glazebrook, 2005). Still, it is effective against adapted powdery mildews if stimulated constitutively, artificially or systemically (Ellis et al., 2002a,b; Stein et al., 2008; Kuhn et al., 2016), but usually either suppressed or failed to be elicited by virulent powdery mildews (Zimmerli et al., 2004; Antico et al., 2012). By contrast, non-adapted powdery mildews trigger JA/ET-dependent plant defense (Zimmerli et al., 2004). Remarkably, GO term overrepresentation, microarray analysis and results of qRT-PCR assays indicated enhanced induction of mRNAs associated with JA/ET signaling in *mlo2 mlo6 mlo12* vs. Col-0 plants (Figure 3, Figure S3, Table 1, Table S2). We detected a statistically significant overaccumulation of the JA-responsive *PDF1.2, VSP2*, and *LOX2* transcripts in the *mlo2 mlo6 mlo12* genotype upon *G. orontii* challenge already at a very early time point after inoculation (8 hpi; Figure 3, Figure S3). This finding resembles the situation of the Arabidopsis-*Bgh* non-host interaction, where *PDF* gene expression also was more strongly induced at early time points after inoculation than in a compatible interaction (Zimmerli et al., 2004). This commonality further corroborates potential mechanistic overlap between NHR and *mlo* resistance. The JA-induced genes *PDF1.2* and *VSP2, LOX2* are responsive to two different branches of JA signaling that depend on the transcription factors (TFs) ORA59/ERF1 and MYC2, respectively (Lorenzo et al., 2004; Zhu et al., 2011; Pieterse et al., 2012; Vos et al., 2013; Wasternack and Hause, 2013). This fact may explain the differential expression kinetics of *PDF1.2* on the one hand and *VSP2* and *LOX2* on the other hand observed following challenge with *G. orontii* (Figure 3, Figure S3E). Together with JA/ET-responsive genes, also *ORA59, ERF1* and *ERF2* mRNAs accumulated to higher levels in *mlo2 mlo6 mlo12* plants vs. Col-0. All three TFs are positive regulators of JA and/or ET-responsive defense gene expression and in turn induced by JA/ET treatment [Table 1; (Pré et al., 2008); Genevestigator (Hruz et al., 2008); Arabidopsis eFP Browser (Winter et al., 2007)]. However, in contrast to *PDF1.2* transcripts, *ORA59* mRNA levels were significantly higher in the *mlo* triple mutant than in the wild type, where it increased only at later stages (12 hpi; Figure 3, Figure S3). Moreover, *MYC2* was not induced to higher levels in the triple mutant, which together might suggest that early *PDF1.2, VSP2* and *LOX2* expression during infection is regulated at least partially independently from an induced expression of these two TFs. Consequently, despite the accumulation of *ORA59, ERF1* and *ERF2* mRNAs, distinct factors might contribute to regulation of JA/ET-responsive genes in the context of powdery mildew challenge of *mlo2 mlo6 mlo12* plants. Alternatively, the increase in *PDF1.2, VSP2* and *LOX2* transcript levels in the *mlo2 mlo6 mlo12* triple mutant might be due to constitutively elevated protein levels of these TFs.

Three findings suggest that the expression of JA/ET responsive genes in the *mlo2 mlo6 mlo12* mutant infected with *G. orontii* occurs independently of canonical JA/ET signaling: First, ET and JA levels are unaltered in *mlo* triple mutant plants after *G. orontii* inoculation (Figure 5). Second, the *mlo2 mlo6 mlo12 ora59* mutant does not show altered resistance to *G. orontii* (Figure 4). Third, prototypical ET/JA signaling components are dispensable for *mlo2*-mediated resistance (Consonni et al., 2006). Developmentally controlled enhanced SA levels in *mlo2* single and *mlo2 mlo6 mlo12* triple mutant plants have been reported previously (Consonni et al., 2006) and were confirmed by our results (Figure 5). However, we could not detect altered SA levels after inoculation with *G. orontii* (Figure 5). This finding is consistent with previous analysis of a barley *mlo* mutant, which also lacked alterations in SA levels following powdery mildew (*Bgh*) challenge (Hückelhoven et al., 1999). It is therefore unlikely that antagonistic (Spoel et al., 2003; Ndamukong et al., 2007; Zander et al., 2010, 2012, 2014; Van der Does et al., 2013) or synergistic/reduced antagonistic (Mur et al., 2006; Liu et al., 2016) effects of SA might impact JA/ET-related gene expression in *mlo* triple mutant plants during fungal infection. In fact, interference with SA abundance and NONEXPRESSER OF PR GENES 1 (NPR)1-mediated SA signaling, responsible for SA/JA antagonism (Spoel et al., 2003), merely suppresses premature senescence of *mlo2* plants, uncoupling this phenotype from penetration resistance (Consonni et al., 2006). In conclusion, *mlo*-mediated resistance has so far not been shown to depend on canonical defense hormone signaling (Consonni et al., 2006). Whether or not a non-canonical induction of JA/ET-related defense gene expression is required for *mlo2 mlo6 mlo12*-mediated resistance remains to be investigated. This might involve transcriptional activation of JA/ET-related genes *via* non- COI1-dependent derepression of JA signaling, possibly including components of SA signaling such as NPR3 and NPR4 and degradation of JAZ (JASMONATE ZIM DOMAIN) proteins, which act as repressors of JA-signaling (Liu et al., 2016).

### Tryptophan-derived antimicrobials are dispensable for *mlo2 mlo6 mlo12*-mediated resistance

Tryptophan-derived antimicrobials such as camalexin and indole glucosinolates contribute to *mlo2*-mediated resistance (Consonni et al., 2010). In line with this finding, we discovered transcripts of biosynthetic enzymes for tryptophan-derived antimicrobials as well as levels of camalexin and the indole glucosinolate 4MI3G (hyper-)accumulated in *G. orontii*-inoculated leaves of *mlo2 mlo6 mlo12* and *mlo2 mlo6 mlo12 pen2* plants, respectively (Figures 7, 8). Remarkably, elevated accumulation of 4MI3G in response to *G. orontii*, but not upon *Bgh* challenge, required in addition to the *pen2* the *mlo2 mlo6 mlo12* mutations. Consequently, in contrast to non-adapted *Bgh*, the adapted fungus either fails to elicit or actively suppresses 4MI3G biosynthesis in wild type plants, as discussed above for transcript accumulation of many defense-related genes. A key role for MLO proteins in defense suppression by adapted powdery mildew pathogens has been previously suggested (Panstruga, 2005). Even if true, it still remains unclear whether the fungus exerts the proposed defense suppression directly *via* the MLO proteins (e.g., prior to host cell entry through manipulation of MLO activity by apoplastic effector proteins), or whether the presence of the MLO proteins is primarily a prerequisite for successful host cell entry and the observed defense suppression merely a secondary effect (e.g., *via* effector proteins secreted by the fungal haustorium).

Surprisingly, loss of function of CYP79B2/CYP97B3, completely depleting the plant for tryptophan-derived antimicrobials (Zhao et al., 2002; Glawischnig et al., 2004), did not interfere with penetration resistance of the *mlo* triple mutant (Figure 9). This is noteworthy since similar to the PEN proteins, CYP79B2/CYP97B3 contribute to resistance against adapted and non-adapted powdery mildews and are required for *mlo2*-mediated resistance against *G. orontii* (Consonni et al., 2010). However, abrogation of CYP79B2/CYP79B3 function likely has a broader effect on the plant defense arsenal than loss of PEN activity. In Arabidopsis, biosynthesis of several defense compounds, such as camalexin (Ahuja et al., 2012), indole glucosinolates (Zhao et al., 2002) and indolic cyanogenic metabolites (e.g., 4-hydroxyindole-3-carbonyl nitrile, 4-OH-ICN; Rajniak et al., 2015) rely on CYP79B2/CYP79B3 activity. The fact that all these antimicrobial metabolites are dispensable for full immunity in the *mlo2 mlo6 mlo12* mutant suggests the involvement of additional highly potent and possibly yet undiscovered defense pathways in Arabidopsis.

## Author contributions

MA, YB, CC, HK, MK, JL, CM, and RP conceived experiments. MA, KB, PB, CC, HK, MK, JL, CM, DM, TR, and EVLvT. performed experiments and analyzed data. IF analyzed data. SB and UC provided expertise and feedback with the FAIRE experiments. HK, JL, and RP wrote the manuscript, YB, UC, IF, and RP secured funding.

### Conflict of interest statement

The authors declare that the research was conducted in the absence of any commercial or financial relationships that could be construed as a potential conflict of interest.
