# Supplementary material for: Key Components of Different Plant Defense Pathways Are Dispensable for Powdery Mildew Resistance of the Arabidopsis mlo2 mlo6 mlo12 Triple Mutant
Source: Front Plant Sci. 2017 Jun 19;8:1006. doi: 10.3389/fpls.2017.01006 (PMC5475338; doi:10.3389/fpls.2017.01006)
Supplement: Supplementary file 2 [file Table2.pdf]

Key Components of Different Plant Defense Pathways Are Dispensable for Powdery Mildew Resistance of the Arabidopsis *mlo2 mlo6 mlo12* Triple Mutant

Hannah Kuhn, Justine Lorek, Mark Kwaaitaal, Chiara Consonni, Katia Becker, Cristina Micali, Emiel Ver Loren van Themaat, Paweł Bednarek, Tom M. Raaymakers, Michela Appiano, Yuling Bai, Dorothea Meldau, Stephani Baum, Uwe Conrath, Ivo Feussner, and Ralph Panstruga

Table S2. Significantly over/underrepresented GO terms (biological process) among genes induced in *mlo2 mlo6 mlo12* vs Col-0 as analyzed by PANTHER. Defense-related GO terms are marked in green, GO terms related to biosynthesis of tryptophan-derived antimicrobials are marked in blue and GO terms associated with JA/ET signaling are marked in red.

GO biological process	*A. thaliana* – REFLIST (27352 genes)	among 145 induced genes	expected	over/under represented	fold enrichment	P-value
S-glycoside biosynthetic process (GO:0016144)	4	3	0.02	+	> 100	3.07E-03
indole glucosinolate biosynthetic process (GO:0009759)	4	3	0.02	+	> 100	3.07E-03
jasmonic acid and ethylene-dependent systemic resistance (GO:0009861)	14	4	0.07	+	55.03	2.25E-03
induced systemic resistance (GO:0009682)	28	6	0.15	+	41.28	2.23E-05
indole-containing compound biosynthetic process (GO:0042435)	34	6	0.18	+	33.99	6.97E-05
defense response to fungus, incompatible interaction (GO:0009817)	37	5	0.19	+	26.03	3.71E-03
indole-containing compound metabolic process (GO:0042430)	52	6	0.27	+	22.23	8.26E-04
toxin metabolic process (GO:0009404)	54	5	0.28	+	17.84	2.29E-02
defense response, incompatible interaction (GO:0009814)	132	12	0.69	+	17.51	1.66E-08
response to organonitrogen compound (GO:0010243)	133	11	0.69	+	15.93	3.41E-07
innate immune response (GO:0045087)	238	18	1.24	+	14.57	1.75E-12
sulfur compound biosynthetic process (GO:0044272)	81	6	0.42	+	14.27	1.04E-02
immune response (GO:0006955)	243	18	1.26	+	14.27	2.49E-12
response to chitin (GO:0010200)	108	8	0.56	+	14.27	2.64E-04
response to wounding (GO:0009611)	179	13	0.93	+	13.99	3.42E-08
response to jasmonic acid (GO:0009753)	173	12	0.9	+	13.36	3.56E-07
immune system process (GO:0002376)	274	19	1.42	+	13.36	1.24E-12
response to nitrogen compound (GO:1901698)	208	11	1.08	+	10.19	3.35E-05
response to salicylic acid (GO:0009751)	164	8	0.85	+	9.40	5.86E-03
response to ethylene (GO:0009723)	230	10	1.19	+	8.37	9.14E-04
sulfur compound metabolic process (GO:0006790)	282	12	1.46	+	8.20	7.76E-05
response to bacterium (GO:0009617)	353	15	1.83	+	8.18	1.43E-06
defense response to other organism (GO:0098542)	770	28	4	+	7.00	1.24E-12
response to organic cyclic compound (GO:0014070)	280	10	1.45	+	6.88	5.25E-03
defense response to bacterium (GO:0042742)	281	10	1.46	+	6.85	5.41E-03
defense response to fungus (GO:0050832)	425	15	2.21	+	6.80	1.69E-05
response to fungus (GO:0009620)	490	17	2.54	+	6.68	2.07E-06
response to external biotic stimulus (GO:0043207)	1002	32	5.2	+	6.15	2.87E-13
response to other organism (GO:0051707)	1002	32	5.2	+	6.15	2.87E-13

GO biological process	*A. thaliana* – REFLIST (27352 genes)	among 145 induced genes	expected	over/under represented	fold enrichment	P-value
response to biotic stimulus (GO:0009607)	1043	32	5.41	+	5.91	8.79E-13
defense response (GO:0006952)	1266	37	6.57	+	5.63	1.29E-14
response to external stimulus (GO:0009605)	1278	34	6.63	+	5.12	5.73E-12
response to acid chemical (GO:0001101)	877	22	4.55	+	4.83	2.50E-06
multi-organism process (GO:0051704)	1326	32	6.88	+	4.65	6.06E-10
response to oxygen-containing compound (GO:1901700)	1151	26	5.98	+	4.35	5.94E-07
response to endogenous stimulus (GO:0009719)	1292	27	6.71	+	4.03	1.39E-06
response to organic substance (GO:0010033)	1475	28	7.66	+	3.66	5.30E-06
response to stress (GO:0006950)	2706	51	14.05	+	3.63	1.50E-13
response to chemical (GO:0042221)	2075	38	10.77	+	3.53	1.03E-08
response to hormone (GO:0009725)	1212	21	6.29	+	3.34	2.91E-03
organonitrogen compound metabolic process (GO:1901564)	1398	21	7.26	+	2.89	2.59E-02
response to stimulus (GO:0050896)	4684	67	24.32	+	2.76	2.81E-13
Unclassified (UNCLASSIFIED)	7701	41	39.98	+	1.03	0.00E+00
