# Supplementary material for: Key Components of Different Plant Defense Pathways Are Dispensable for Powdery Mildew Resistance of the Arabidopsis mlo2 mlo6 mlo12 Triple Mutant
Source: Front Plant Sci. 2017 Jun 19;8:1006. doi: 10.3389/fpls.2017.01006 (PMC5475338; doi:10.3389/fpls.2017.01006)
Supplement: Supplementary file 3 [file Table3.pdf]

frontiers

Key Components of Different Plant Defense Pathways Are Dispensable for Powdery Mildew Resistance of the Arabidopsis *mlo2 mlo6 mlo12* Triple Mutant

Hannah Kuhn, Justine Lorek, Mark Kwaaitaal, Chiara Consonni, Katia Becker, Cristina Micali, Emiel Ver Loren van Themaat, Paweł Bednarek, Tom M. Raaymakers, Michela Appiano, Yuling Bai, Dorothea Meldau, Stephani Baum, Uwe Conrath, Ivo Feussner, and Ralph Panstruga

Table S3. Oligonucleotides used for qPCR in this study. Numbers in qFAIRE-PCR oligo names indicate amplicon position relative to the ATG of the corresponding gene.

**qRT-PCR**		
**Oligo**	**Sequence (5'-3')**	**Reference**
At4g26420-F	GAGCTGAAGTGGCTTCCATGAC	Czechowski et al., 2005
At4g26420-R	GGTCCGACATACCCATGATCC	
PDF1.2a-F	TAAGTTTGCTTCCATCATCACCC	Zimmerli et al., 2004
PDF1.2a-R	GTGCTGGGAAGACATAGTTGCAT	
PDF1.2b-F	ACGCTGCTCTTGTTCTCTTTGCA	Zimmerli et al., 2004
PDF1.2b-R	AAGTACCACTTGGCTTCTCGCAC	
LOX2-F	TGAATTGCAAGCTGTTGCTC	R. Birkenbihl, unpublished
LOX2-R	GCAGAAGCTACAAGACCACC	
VSP2-F	ACCGTTGGAAGTTGTGGAAG	M. Schön, unpublished
VSP2-R	CCAAATCAGCCCATTGATCT	
ORA59-F	CTCGCTTCTACAATTTTTATG	Pré et al., 2008
ORA59-R	GGAAACATGTATAGATGTGTAG	
**qFAIRE-PCR**		
**Oligo**	**Sequence (5'-3')**	**Reference**
ACTIN2-OC+35-F	GGCTGAGGCTGGTC	S. Baum, unpublished
ACTIN2-OC+35-R	AACCAGCCTTCACCACC	
PDF1.2a-OC-93-F	CTTCTTTTGTGGCTACGGG	this study
PDF1.2a-OC-93-R	GTGGGTTACTTCTACTTGTGCC	
PDF1.2a-OC-288-F	CAACTTGTGTAACTGGCTTGG	this study
PDF1.2a-OC-288-R	CTACCGTTCACGGGCG	
PDF1.2a-OC-384-F	CTCTACGAAAAGTTGTTTGTTCG	this study
PDF1.2a-OC-384-R	GAGTCTCGCGGTTAGGACAAG	
PDF1.2a-OC-476-F	CTTGTTCTGCAAACTTGGCG	this study
PDF1.2a-OC-476-R	GCACCCCTAGACAGCGTTG	
PDF1.2a-OC-543-F	CATTTCAGTAAGGTGTGTCCCAG	this study
PDF1.2a-OC-543-R	CGCCAAGTTTGCAGAACAAG	
PDF1.2a-OC-633-F	CTGCCCTCTCCGTTCCTCC	this study
PDF1.2a-OC-633-R	GGGTCCATTTGCTCTC	
PDF1.2a-OC-817-F	TACGAAACCTTTAGCTACTACA	this study
PDF1.2a-OC-817-R	GTCTGACCGCAAGTGAGAGGC	
PDF1.2b-OC-67-F	CTCGAGAAGGCGTACGTGG	this study
PDF1.2b-OC-67-R	GTGAAGAAAAGCTTAAGAGCAACC	

Oligo	Sequence (5'-3')	Reference
PDF1.2b-OC-186-F	GCTTAGGTTACTAGTGAGAGAG	this study
PDF1.2b-OC-186-R	CACCACGTACGCCTTCTCG	
PDF1.2b-OC-309-F	CACTTTTCCTAACTGTGAAACTCTG	this study
PDF1.2b-OC-309-R	CTGCTTTGGCTTTGCGGG	
PDF1.2b-OC-374-F	GCACAATGAAAGCTTACACTTAC	this study
PDF1.2b-OC-374-R	CTTAACCAAGCCAAGTTACACAAG	
PDF1.2b-OC-457-F	TGGCTCAACGGAAATTTGACC	this study
PDF1.2b-OC-457-R	CGAAAACCTTGAGTAAGTGTAAG	
PDF1.2b-OC-602-F	CGTAGGTGTTTCCTGGTAAAG	this study
PDF1.2b-OC-602-R	ACTCGACGGTTTGGTTGC	
PDF1.2b-OC-685-F	AGGTTTGTTTACAGCAACTTAGC	this study
PDF1.2b-OC-685-R	CCTTACTTTACCAGGAAACACC	
PDF1.2b-OC-813-F	CACGACTGTTTCCGGCC	this study
PDF1.2b-OC-813-R	GGTCGTGGCTGATTAGGG	
PDF1.2b-OC-995-F	TGGGATTGGGGAAGCAAG	this study
PDF1.2b-OC-995-R	CTGGTGTAGCTCGTCCC	
