# Supplementary material for: Key Components of Different Plant Defense Pathways Are Dispensable for Powdery Mildew Resistance of the Arabidopsis mlo2 mlo6 mlo12 Triple Mutant
Source: Front Plant Sci. 2017 Jun 19;8:1006. doi: 10.3389/fpls.2017.01006 (PMC5475338; doi:10.3389/fpls.2017.01006)
Supplement: Supplementary file 4 [file Image1.pdf]

Key Components of Different Plant Defense Pathways Are Dispensable for Powdery Mildew Resistance of the Arabidopsis *mlo2 mlo6 mlo12* Triple Mutant

Hannah Kuhn, Justine Lorek, Mark Kwaaitaal, Chiara Consonni, Katia Becker, Cristina Micali, Emiel Ver Loren van Themaat, Paweł Bednarek, Tom M. Raaymakers, Michela Appiano, Yuling Bai, Dorothea Meldau, Stephani Baum, Uwe Conrath, Ivo Feussner, and Ralph Panstruga

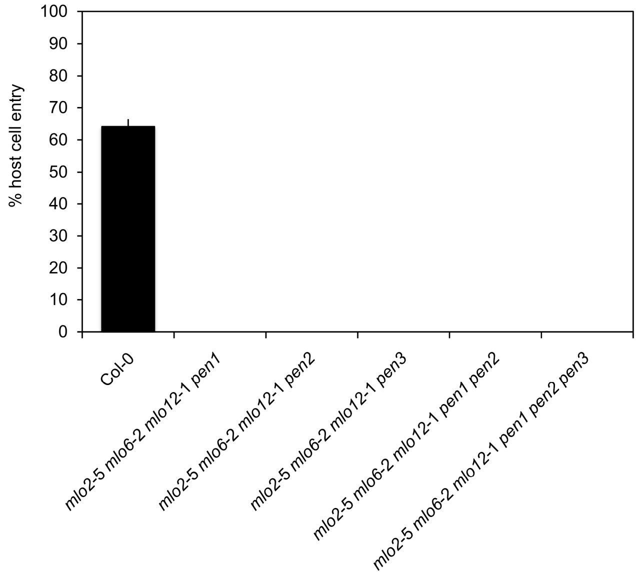

Figure S1. *mlo2 mlo6 mlo12*-mediated resistance functions independently of the PEN1 and PEN2/PEN3 defense pathways upon inoculation with *O. neolycopersici*. 4-5-week-old plants of the indicated genotypes were inoculated with *O. neolycopersici* and % host cell entry was evaluated at 48 hpi. Data represent means of 3 biological replicates (plants) +/- SD.
