# Supplementary material for: Key Components of Different Plant Defense Pathways Are Dispensable for Powdery Mildew Resistance of the Arabidopsis mlo2 mlo6 mlo12 Triple Mutant
Source: Front Plant Sci. 2017 Jun 19;8:1006. doi: 10.3389/fpls.2017.01006 (PMC5475338; doi:10.3389/fpls.2017.01006)
Supplement: Supplementary file 5 [file Image2.pdf]

Key Components of Different Plant Defense Pathways Are Dispensable for Powdery Mildew Resistance of the Arabidopsis *mlo2 mlo6 mlo12* Triple Mutant

Hannah Kuhn, Justine Lorek, Mark Kwaaitaal, Chiara Consonni, Katia Becker, Cristina Micali, Emiel Ver Loren van Themaat, Paweł Bednarek, Tom M. Raaymakers, Michela Appiano, Yuling Bai, Dorothea Meldau, Stephani Baum, Uwe Conrath, Ivo Feussner, and Ralph Panstruga

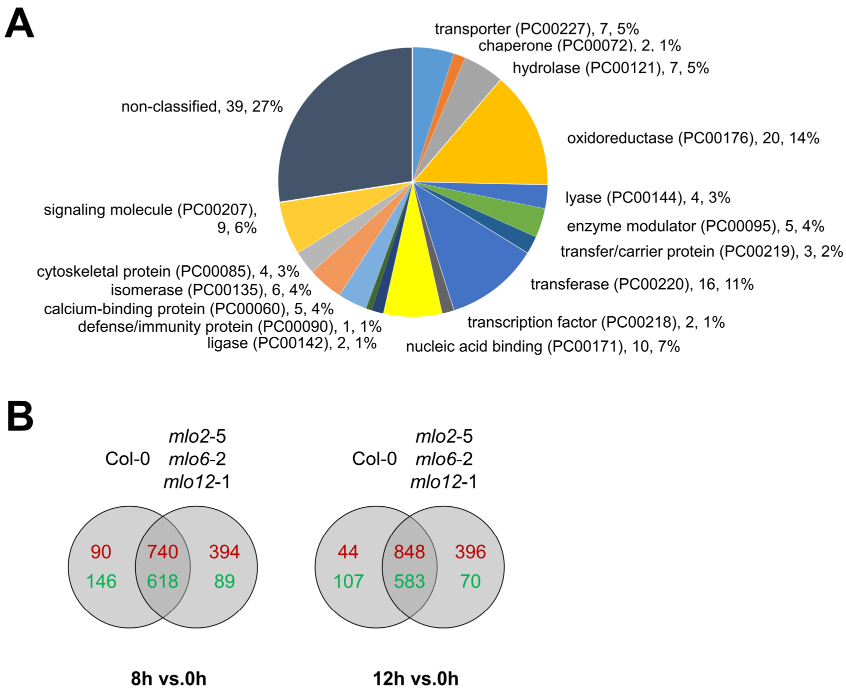

Figure S2. *mlo2 mlo6 mlo12* contributes to accelerated transcript accumulation during *G. orontii* infection. 4-5-week-old *mlo2*-5 *mlo6*-2 *mlo12*-1 and Col-0 plants were inoculated with *G. orontii* and sampled prior to inoculation (0 h, non-challenged) or at 8 and 12 hpi for comparative transcriptome analysis using the Affymetrix ATH1 GeneChip. All samples were analyzed in triplicates. (A) Functional classification of genes upregulated in *mlo2 mlo6 mlo2* vs Col-0. after *G. orontii* inoculation based on PANTHER protein classes. (B) Venn diagrams displaying the number of genes with statistically significantly ($P \leq 0.05$) increased ($\geq$ 2-fold; red) or decreased ($\leq$ 0.5-fold; green) transcript abundance at 8 hpi (left) or 12 hpi (right) vs. 0 h in *mlo2 mlo6 mlo12* in comparison with Col-0.
