# Supplementary material for: Key Components of Different Plant Defense Pathways Are Dispensable for Powdery Mildew Resistance of the Arabidopsis mlo2 mlo6 mlo12 Triple Mutant
Source: Front Plant Sci. 2017 Jun 19;8:1006. doi: 10.3389/fpls.2017.01006 (PMC5475338; doi:10.3389/fpls.2017.01006)
Supplement: Supplementary file 7 [file Image4.pdf]

Key Components of Different Plant Defense Pathways Are Dispensable for Powdery Mildew Resistance of the Arabidopsis *mlo2 mlo6 mlo12* Triple Mutant

Hannah Kuhn, Justine Lorek, Mark Kwaaitaal, Chiara Consonni, Katia Becker, Cristina Micali, Emiel Ver Loren van Themaat, Paweł Bednarek, Tom M. Raaymakers, Michela Appiano, Yuling Bai, Dorothea Meldau, Stephani Baum, Uwe Conrath, Ivo Feussner, and Ralph Panstruga

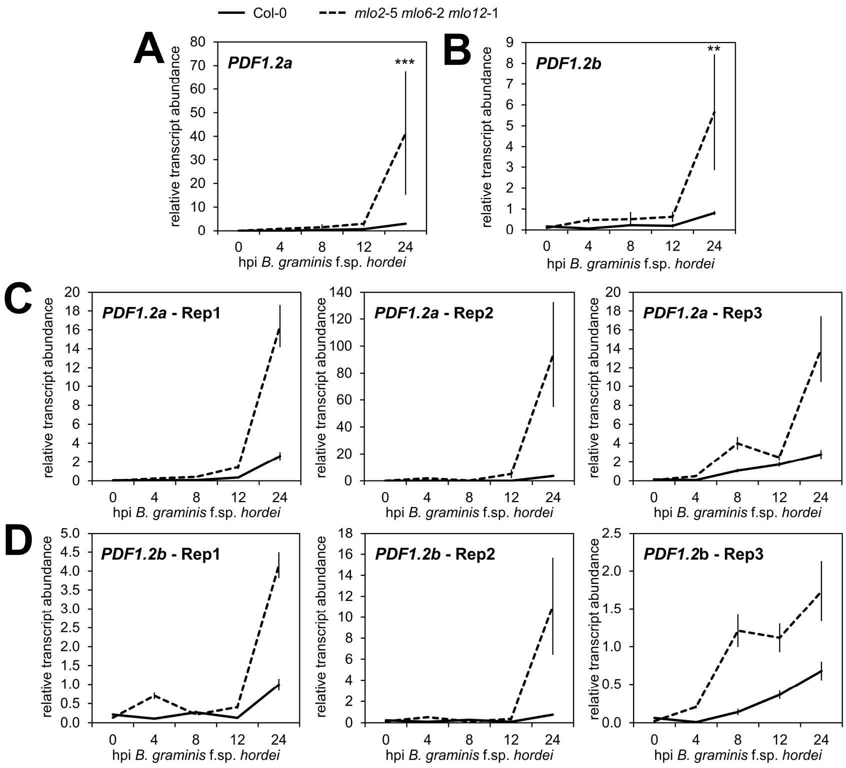

Figure S4. Transcripts of *PDF1.2a* and *PDF1.2b* accumulate more rapidly and to higher levels in *mlo2 mlo6 mlo12* vs. Col-0 after inoculation with *B. graminis* f.sp. *hordei*. qRT-PCR analysis of *PDF1.2a* and *PDF1.2b* transcript accumulation in leaves of 4-5-week-old Col-0 (solid line) and *mlo2 mlo6 mlo12* (dashed line) plants inoculated with *B. graminis* f.sp. *hordei* and sampled prior to inoculation (0 h) or at 4, 8, 12 and 24 hpi. (A and B) Means +/- SE of three independent biological replicates are shown. Asterisks indicate a statistically significant difference from Col-0 (*** $P \leq 0.01$, ** $P \leq 0.01$*, $P \leq 0.05$, GLM). (C and D) Means +/- SD of three technical replicates within individual independent biological replicates are shown. Gene expression was normalized to the transcript levels of the reference gene *At4g26420*.
