# Supplementary material for: Key Components of Different Plant Defense Pathways Are Dispensable for Powdery Mildew Resistance of the Arabidopsis mlo2 mlo6 mlo12 Triple Mutant
Source: Front Plant Sci. 2017 Jun 19;8:1006. doi: 10.3389/fpls.2017.01006 (PMC5475338; doi:10.3389/fpls.2017.01006)
Supplement: Supplementary file 8 [file Image5.pdf]

Key Components of Different Plant Defense Pathways Are Dispensable for Powdery Mildew Resistance of the Arabidopsis *mlo2 mlo6 mlo12* Triple Mutant

Hannah Kuhn, Justine Lorek, Mark Kwaaitaal, Chiara Consonni, Katia Becker, Cristina Micali, Emiel Ver Loren van Themaat, Paweł Bednarek, Tom M. Raaymakers, Michela Appiano, Yuling Bai, Dorothea Meldau, Stephani Baum, Uwe Conrath, Ivo Feussner, and Ralph Panstruga

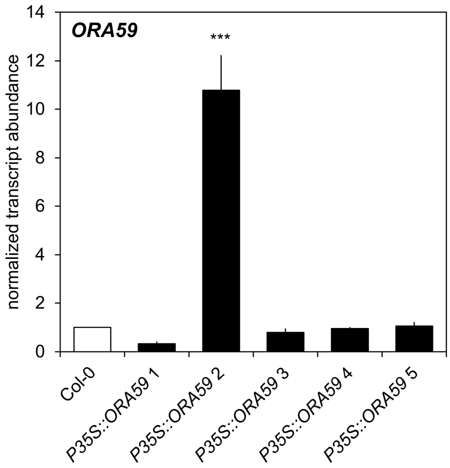

Figure S5. Transcript abundance of *ORA59* is increased in transgenic line 2 of Col-0 stably expressing ORA59 under control of the 35S cauliflower mosaic virus (CMV) promoter. qRT-PCR analysis of *ORA59* transcript accumulation in leaves of Col-0 and five different 4-5-week-old transgenic *P35S::ORA59* overexpressing lines (Pré et al., 2008). Means +/- SE of three independent biological replicates are shown. Asterisks indicate a statistically significant difference from Col-0 (*** $P \leq 0.01$, GLM). Gene expression was first normalized to the transcript levels of the reference gene *At4g26420* and afterwards to values obtained for Col-0.
