# Supplementary material for: Key Components of Different Plant Defense Pathways Are Dispensable for Powdery Mildew Resistance of the Arabidopsis mlo2 mlo6 mlo12 Triple Mutant
Source: Front Plant Sci. 2017 Jun 19;8:1006. doi: 10.3389/fpls.2017.01006 (PMC5475338; doi:10.3389/fpls.2017.01006)
Supplement: Supplementary file 9 [file Image6.pdf]

Key Components of Different Plant Defense Pathways Are Dispensable for Powdery Mildew Resistance of the Arabidopsis *mlo2 mlo6 mlo12* Triple Mutant

Hannah Kuhn, Justine Lorek, Mark Kwaaitaal, Chiara Consonni, Katia Becker, Cristina Micali, Emiel Ver Loren van Themaat, Paweł Bednarek, Tom M. Raaymakers, Michela Appiano, Yuling Bai, Dorothea Meldau, Stephani Baum, Uwe Conrath, Ivo Feussner, and Ralph Panstruga

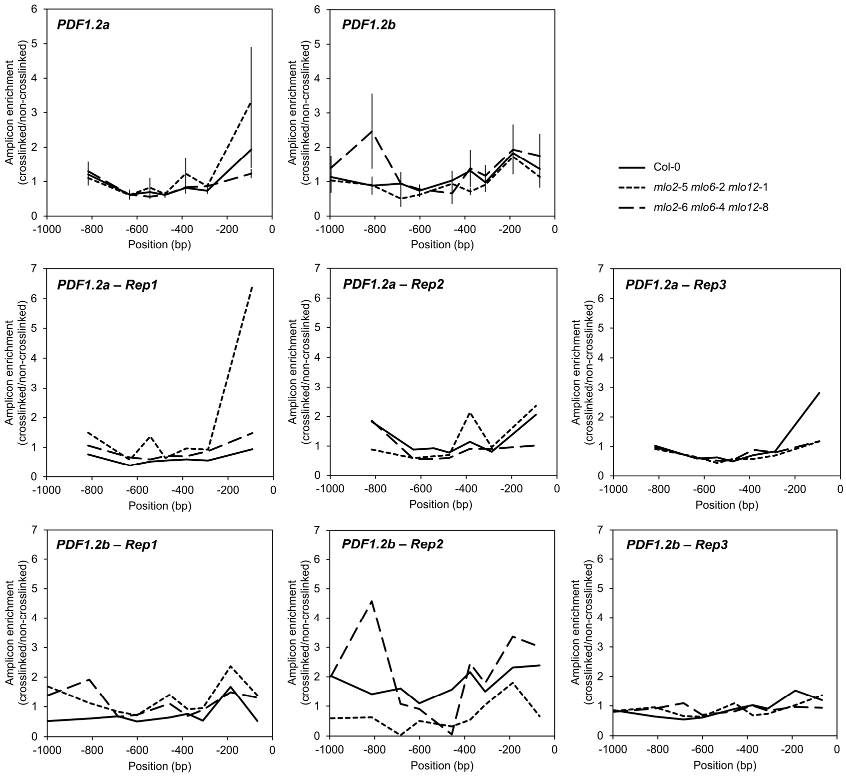

Figure S6. The *mlo2 mlo6 mlo12* mutations do not coincide with increased accessibility of the *PDF1.2a* and *PDF1.2b* promoters. FAIRE and qPCR analysis of the *PDF1.2a* and *PDF1.2b* promoters. Leaves of 4-5-week-old Col-0 (solid line) and *mlo2 mlo6 mlo12* (dashed lines) plants were sampled and crosslinked. Isolated DNA was sonicated and abundance of amplicons within the promoters of *PDF1.2a* and *PDF1.2b* was measured. Amplicon enrichment of crosslinked vs. de-crosslinked DNA of the same sample was calculated after normalization to an amplicon within the ORF of the reference gene *ACTIN2* (*At3g18780*). The central amplicon positions are given as bp upstream of ATG. Means +/- SE of three independent biological replicates (upper row) and means +/- SD of three technical replicates within individual independent biological replicates are shown (lower rows). No statistically significant differences from Col-0 were detected by GLM.
