# Supplementary material for: Key Components of Different Plant Defense Pathways Are Dispensable for Powdery Mildew Resistance of the Arabidopsis mlo2 mlo6 mlo12 Triple Mutant
Source: Front Plant Sci. 2017 Jun 19;8:1006. doi: 10.3389/fpls.2017.01006 (PMC5475338; doi:10.3389/fpls.2017.01006)
Supplement: Supplementary file 10 [file Image7.pdf]

Key Components of Different Plant Defense Pathways Are Dispensable for Powdery Mildew Resistance of the Arabidopsis *mlo2 mlo6 mlo12* Triple Mutant

Hannah Kuhn, Justine Lorek, Mark Kwaaitaal, Chiara Consonni, Katia Becker, Cristina Micali, Emiel Ver Loren van Themaat, Paweł Bednarek, Tom M. Raaymakers, Michela Appiano, Yuling Bai, Dorothea Meldau, Stephani Baum, Uwe Conrath, Ivo Feussner, and Ralph Panstruga

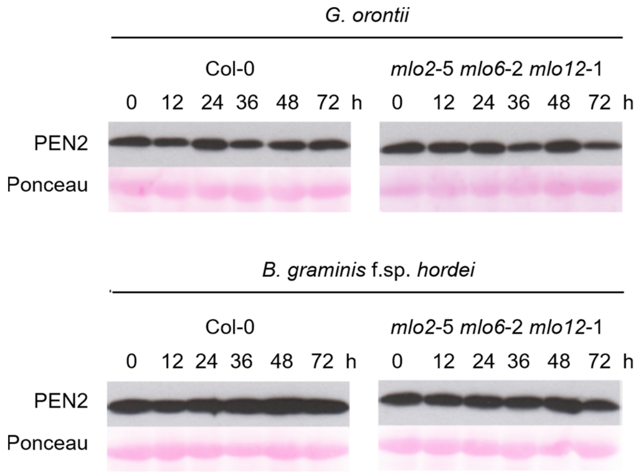

Figure S7. PEN2 does not differentially accumulate in *mlo2 mlo6 mlo12* mutant plants after inoculation with *G. orontii* or *B. graminis* f.sp. *hordei*. Total protein extracts from 4-5-week-old plants inoculated with *G. orontii* or *B. graminis* f.sp. *hordei* for the indicated time points were separated by SDS-PAGE and probed with α-PEN2 antiserum. Ponceau staining served as a loading control. The experiment was repeated once with similar results.
